# Impact of New Cardiovascular Events on Quality of Life and Hospital Costs in People With Cardiovascular Disease in the United Kingdom and United States

**DOI:** 10.1161/JAHA.123.030766

**Published:** 2023-09-26

**Authors:** Juliana Nga Man Lui, Claire Williams, Mi Jun Keng, Jemma C. Hopewell, Emily Sammons, Fang Chen, Alastair Gray, Louise Bowman, Sir Martin J. Landray, Borislava Mihaylova, the REVEAL Collaborative Group

**Affiliations:** ^1^ Health Economics Research Centre, Nuffield Department of Population Health University of Oxford Oxford United Kingdom; ^2^ Clinical Trial Service Unit and Epidemiological Studies Unit Nuffield Department of Population Health University of Oxford United Kingdom; ^3^ Medical Research Council Population Health Research Unit, Nuffield Department of Population Health University of Oxford United Kingdom; ^4^ Health Economics and Policy Research Unit, Wolfson Institute of Population Health Queen Mary University of London United Kingdom

**Keywords:** cardiovascular diseases, health care costs, quality of life, secondary prevention, United Kingdom, United States, Cardiovascular Disease, Secondary Prevention, Quality and Outcomes, Health Services, Cost-Effectiveness

## Abstract

**Background:**

Despite optimized risk factor control, people with prior cardiovascular disease remain at high cardiovascular disease risk. We assess the immediate‐ and longer‐term impacts of new vascular and nonvascular events on quality of life (QoL) and hospital costs among participants in the REVEAL (Randomized Evaluation of the Effects of Anacetrapib Through Lipid Modification) trial in secondary prevention.

**Methods and Results:**

Data on demographic and clinical characteristics, health‐related quality of life (QoL: EuroQoL 5‐Dimension‐5‐Level), adverse events, and hospital admissions during the 4‐year follow‐up of the 21 820 participants recruited in Europe and North America informed assessments of the impacts of new adverse events on QoL and hospital costs from the UK and US health systems' perspectives using generalized linear regression models. Reductions in QoL were estimated in the years of event occurrence for nonhemorrhagic stroke (−0.067 [United Kingdom], −0.069 [US]), heart failure admission (−0.072 [United Kingdom], −0.103 [US]), incident cancer (−0.064 [United Kingdom], −0.068 [US]), and noncoronary revascularization (−0.071 [United Kingdom], −0.061 [US]), as well as in subsequent years following these events. Myocardial infarction and coronary revascularization (CRV) procedures were not found to affect QoL. All adverse events were associated with additional hospital costs in the years of events and in subsequent years, with the highest additional costs in the years of noncoronary revascularization (£5830 [United Kingdom], $14 133 [US Medicare]), of myocardial infarction with urgent CRV procedure (£5614, $24722), and of urgent/nonurgent CRV procedure without myocardial infarction (£4674/£4651 and $15 251/$17 539).

**Conclusions:**

Stroke, heart failure, and noncoronary revascularization procedures substantially reduce QoL, and all cardiovascular disease events increase hospital costs. These estimates are useful in informing cost‐effectiveness of interventions to reduce cardiovascular disease risk in secondary prevention.

**Registration:**

URL: https://www.clinicaltrials.gov; Unique identifier: NCT01252953; https://www.Isrctn.com. Unique identifier: ISRCTN48678192; https://www.clinicaltrialsregister.eu. Unique identifier: 2010‐023467‐18.

**Nonstandard Abbreviations and Acronyms**
CRV: coronary revascularization
GLM: generalized linear model
REVEAL: Randomized Evaluation of the Effects of Anacetrapib Through Lipid Modification

**Clinical Perspective**

**What Is New?**

- The study estimates the impacts of new major cardiovascular events on quality of life and hospital care costs in contemporary patients with well‐managed secondary cardiovascular disease from the perspectives of the UK and US health systems.
- In the year of event occurrence, nonhemorrhagic stroke, heart failure admission, and noncoronary revascularization were associated with the largest reductions in quality of life, whereas no quality of life reductions were observed for myocardial infarction and coronary revascularization.
- Coronary and noncoronary revascularization procedures were associated with the largest additional hospital costs.

**What Are the Clinical Implications?**

- The contemporary impacts of new cardiovascular events on quality of life and costs reported in this study could inform cost‐effectiveness evaluations of newer interventions for secondary cardiovascular disease prevention in the United Kingdom and United States.

Cardiovascular disease (CVD) is the leading cause of morbidity and mortality worldwide, accounting for a third of deaths globally,^1^ an eighth of health care expenditure,^2^ and significant reduction in quality of life (QoL).^3^, ^4^ Despite improved risk factor management with the use of statin, antihypertensive, and antithrombotic therapies, people with prior CVD remain at high risk of further cardiovascular events.^5^, ^6^ Thus, new medications to reduce this risk are continually being sought.^7^ Decision‐analytic models are frequently used for evaluating interventions. These models can combine the impacts of treatment across a range of outcomes to assess net health effects and cost‐effectiveness over the long term.^8^, ^9^ Quality‐adjusted life years (QALYs), a combined measure of life expectancy and health‐related QoL, is a widely adopted metric used to quantify effectiveness of interventions.^10^, ^11^ Quantifying the impact of new treatments on QoL‐adjusted survival and the use and costs of health care is important to justify their costs.

Cardiovascular events such as myocardial infarction (MI), stroke, and heart failure have been associated with substantial long‐term impacts on QoL^3^, ^4^ and health care costs.^12^, ^13^ However, improved lifestyle and environmental factors, as well as better CVD management, have been linked with reduced morbidity, disease severity, and mortality^14^ and may also have reduced the disease impacts on QoL and health care costs reported in earlier studies. As QoL and hospital costs associated with disease events are crucial elements in cost‐effectiveness and policy assessments, up‐to‐date estimates are required.

The REVEAL (Randomized Evaluation of the Effects of Anacetrapib Through Lipid Modification) trial^15^ assessed effects of anacetrapib 100 mg daily on the risk of major coronary events among 30 449 patients with prior atherosclerotic vascular disease, randomized in 431 sites in Europe, North America, and China. Participants were eligible if they were aged 50 years or older and previously had a MI, cerebrovascular atherosclerotic disease, peripheral artery disease, or diabetes with symptomatic coronary heart disease before entering the trial. Participants were well managed on established cardiovascular therapies and their baseline characteristics and adverse events during the study were well characterized. REVEAL study results have been previously reported.^16^, ^17^ We present estimates of the impacts of key vascular and nonvascular events during study follow‐up on participants' QoL and hospital care costs.

## Methods

The data underlying this article are available in the article and in its online supplementary material. The Nuffield Department of Population Health data access policy is available at https://www.ndph.ox.ac.uk/data‐access.

A total of 21 820 REVEAL participants, recruited in Europe and North America from 2011 to 2013 and followed for an average of 4 years until 2017, contributed to the main analyses in the current study. Data on the 8629 participants recruited in China were excluded from the main study analyses because of large differences in hospitalization rates for events and much longer inpatient stays, suggesting different hospital care patterns compared with the other study regions. Previous studies have also reported that EuroQoL 5‐Dimension (EQ‐5D) scores exhibit a larger ceiling effect in a Chinese population suggesting cultural influences limiting comparisons with Western populations.^18^ We therefore report QoL reductions associated with cardiovascular effects among Chinese participants in a separate scenario analysis.

### Key Disease Events

After randomization, follow‐up assessments in the study were conducted at 2 months, 6 months, and every 6 months thereafter, and information was sought for serious adverse events including those resulting in hospital admissions. Key disease events considered in the present study were postrandomization MI, nonhemorrhagic stroke, urgent coronary revascularization (CRV) procedure, nonurgent CRV procedure, hospitalization for heart failure, noncoronary revascularization procedure, incident cancer, incident diabetes, vascular death, and nonvascular death. All reports of possible such events, except diabetes, were centrally adjudicated by clinicians without prior knowledge of the treatment allocations according to prespecified criteria.^15^

### Health‐Related QoL

Participants' health‐related QoL was assessed using the EQ‐5D‐5‐Level (5L) questionnaire,^19^ administered by trained health care professionals at randomization and at final follow‐up visit in the study. The EQ‐5D‐5L comprises 5 dimensions: mobility, self‐care, usual activities, pain/discomfort, and anxiety/depression, each rated on a scale from 1 to 5, corresponding to no problems, slight problems, moderate problems, severe problems, and extreme problems. Overall QoL utility scores were derived for all contributing study participants using the UK National Institute for Health and Care Excellence decision support unit scoring algorithm (based on the Policy Research Unit in Economic Methods of Evaluation in Health and Social Care Interventions [EEPRU] survey data) for the UK perspective^20^ and, separately, the US EQ‐5D‐5L value set for the US perspective,^21^ where a QoL utility score of 1 corresponds to full health, 0 to health state equivalent to death, and negative values to health states worse than death. In a scenario analysis, the QoL for participants recruited in China was also assessed using the Chinese EQ‐5D‐5L value set.^22^

Previous research has indicated that an individual's QoL was affected most by recent experiences of adverse events with impact on QoL diminishing thereafter.^23^ Therefore, to evaluate the immediate and longer‐term impacts of new key disease events on QoL, hierarchical temporal event history categories were generated for each participant with respect to the time (in years) between the date of the last occurrence of the respective event during the study follow‐up and the date of participants' QoL measurement at end of study. These temporal categories were *no new event*, or new event occurred *within 1 year* (≤*1 year*), *within 1 to 2 years*, *within 2 to 3 years*, or *more than 3 years* before the QoL measure at end of study.

### Hospital Costs

For each study participant, hospital episodes were defined using the hospital admissions data during follow‐up; hospital admissions with overlapping lengths of stay were combined. For UK perspective, hospital episodes were mapped to Healthcare Resource Groups^24^ and costed using National Health Service reference cost 2015 to 2016,^25^ then inflated to year 2019 using National Health Service cost index.^26^ For the US perspective, hospital episodes were mapped to diagnosis‐related groups^27^ and costed using Medicare payment data 2016,^27^ inflated to year 2019 using US gross domestic product index,^28^ and a 20% payment uplift was included for professional fees.^29^ Hospital episode costs incurred by each patient were aggregated into annual periods from randomization to the end of follow‐up.

To evaluate the immediate and longer‐term impacts of new key disease events on hospital costs, hierarchical temporal event history categories were generated for each year of follow‐up in the study with respect of the first occurrence of events of each type since randomization. These temporal categories were no new event, new event occurred *in current year* (≤*1 year*), *1 to 2 years ago*, *2 to 3 years ago*, and *more than 3 years ago*.

### Statistical Analysis

#### Specification of Regression Models

The generalized linear model (GLM) framework was adopted to facilitate modeling the QoL and annual hospital costs. For the QoL analysis, QoL utility at final follow‐up was modeled using the GLM with a Gaussian family distribution and linear link function, adjusting for participant QoL at randomization. Study participants who died before QoL measurement or did not provide QoL measure at study end were excluded from QoL analysis. For the hospital cost analysis, in view of the substantial proportion of annual periods without hospital care costs, the 2‐part (part 1: logistic model for likelihood of incurring any cost, part 2: magnitude of cost, conditional on incurring cost) GLM specifications with Gaussian, Poisson, and gamma family distributions, in conjunction with identity and log link functions, were compared. Tests of appropriateness of distribution and link function and model fit statistics guided the choice of the final GLM specifications. Urgent and nonurgent CRV events and hospital admissions for heart failure always occurred in hospital. Therefore, these events' respective annual periods of occurrence were excluded from the estimation of the first part of the 2‐part models with the probability of incurring hospital costs then assumed certain (100%). SEs in the hospital cost regression model were adjusted for clustering by participant to account for correlation between the multiple annual periods for individual participants during follow‐up.

In both the QoL and hospital cost regression models, adjustments were considered for participants' demographic and clinical characteristics and hierarchical temporal event history categories. The small amount of missing data for covariates was imputed with mean values by sex, smoking status, and trial arm. Backward and forward covariate selections were carried out on all events of interest with their hierarchical temporal event history categories and the full set of participant characteristics. With the exception of covariates for age and sex that were always retained, only covariates statistically significant at 1% level were retained in final models. Interactions between co‐occurring events were tested when such co‐occurrences exceeded 5% of the total combined number of contributing events. The F‐test (at 1% significance level) was used to assess statistical differences in the effects between each 2 consecutive hierarchical temporal event history categories from the most distant temporal category (more than 3 years since the qualifying event) backwards to the most recent category (ie, ≤1 year from event/year of event). Lack of evidence for a statistically significant difference at each step led to combining respective temporal categories and reestimating the model before the next step.

The additional annual hospital costs in year of occurrence of disease events were estimated using the recycled prediction method^30^ with SEs derived using 1000 bootstrap resamples. Further details of the procedures used to select the GLMs and covariates in final models are provided in Data S2.

#### Scenario Analyses

In scenario analyses, the QoL reductions and additional annual hospital costs associated with adverse events were reestimated by only including the UK study participants in analysis from the UK perspective, and separately, only including the participants recruited in North America in the analysis from the US perspective. Furthermore, results from scenario analysis excluding the adjustment for participant baseline QoL were reported (for use in situations when no baseline QoL is available). In a further scenario analysis, the QoL regression model was estimated only among study participants recruited in China and using the Chinese EQ‐5D tariff.^22^

All analyses were conducted using R 3.4.2. *P* values <0.01 were considered to be statistically significant.

The study was approved by Oxfordshire Research Ethics Committee (REC) B (10/H0605/83). All the patients provided written informed consent.

## Results

### Study Participants

At randomization, the mean age across the 21 820 participants contributing to the main analyses was 67 years, 85.7% were men, 62.6% reported history of MI alone, 11.1% cerebrovascular disease alone, 5.7% peripheral artery disease alone; and the remaining participants reported prior disease histories in 2 or more of these categories or none of these conditions (Table 1). Diabetes was reported by 33.7%, heart failure by 5.2%, and atrial fibrillation by 6.7% of participants. Large proportions of participants reported use of antihypertensive (93.4% of participants), antithrombotic (96.6%), and statin (97.2%) therapy. The mean QoL utility of study participants at randomization was 0.84 (SD 0.16) (UK perspective) and 0.88 (SD 0.17) (US perspective).

**Table 1:** Baseline Characteristics of the 21 820 REVEAL Participants Randomized in Europe and North America

	No. (%) or Mean (SD)
Female sex	3110 (14.3%)
Age, y	67.09 (standard deviation 8.3)
Study region	
United Kingdom	8381 (38.4%)
Other European countries	7357 (33.7%)
North America	6082 (27.9%)
Cardiovascular disease	
MI only	13 652 (62.6%)
CEV only	2426 (11.1%)
PAD only	1245 (5.7%)
MI and CEV	1536 (7.0%)
MI and PAD	597 (2.7%)
CEV and PAD	335 (1.5%)
MI, CEV, and PAD	182 (0.8%)
None of MI, CEV, PAD	1847 (8.5%)
Heart failure	1128 (5.2%)
Atrial fibrillation	1457 (6.7%)
Diabetes	7360 (33.7%)
Systolic blood pressure, mm Hg	
<125	8482 (38.9%)
≥125 to <140	6954 (31.9%)
≥140	6384 (29.3%)
Diastolic blood pressure, mm Hg	
<75	8399 (38.5%)
≥75 to <85	7746 (35.5%)
≥85	5675 (26.0%)
Body mass index, kg/m^2^	
<25	3542 (16.2%)
≥25 to <30	9453 (43.3%)
≥30	8785 (40.3%)
Missing	40 (0.2%)
Alcohol status	
Current drinker	9088 (41.6%)
Former drinker	2421 (11.1%)
Never drinker	10 311 (47.3%)
Smoking status	
Current smoker	2774 (12.7%)
Former smoker	10 013 (45.9%)
Never smoker	9033 (41.4%)
Medication use	
Antihypertensives	20 378 (93.4%)
Antithrombotic	21 075 (96.6%)
Amiodarone	344 (1.6%)
Statin (at study entry)	21 215 (97.2%)
Low‐density lipoprotein cholesterol, mmol/L	
<1.4	6804 (31.2%)
≥1.4 to <1.7	6471 (29.7%)
≥1.7	8322 (38.1%)
Missing	223 (1.0%)
High‐density lipoprotein cholesterol, mmol/L	
<0.9	5791 (26.5%)
≥0.9 to <1.1	7209 (33.0%)
≥1.1	8597 (39.4%)
Missing	223 (1.0%)
Triglycerides, mmol/L	
<1.2	8589 (39.4%)
≥1.2 to <1.7	5957 (27.3%)
≥1.7	7051 (32.3%)
Missing	223 (1.0%)
Glomerular filtration rate, mL/min per 1.73 m^2^ *	
<45	18 709 (85.7%)
≥45 to <60	766 (3.5%)
≥60	2122 (9.7%)
Missing	223 (1.0%)
Urine albumin‐to‐creatinine ratio, mg/mmol	
<3	17 251 (79.1%)
≥3 to <30	2817 (12.9%)
≥30	537 (2.5%)
Missing	1215 (5.6%)

CEV indicates cerebrovascular disease; MI, myocardial infarction; PAD, peripheral artery disease; and REVEAL, Randomized Evaluation of the Effects of Anacetrapib Through Lipid Modification.

* The estimated glomerular filtration rate was calculated with the use of the Chronic Kidney Disease Epidemiology Collaboration equation. The missing data were imputed with mean values by sex, smoking status, and trial arm before further analyses.

During the 4 years of study follow‐up, 989 out of 21 820 participants (4.5%) experienced an MI, 735 (3.4%) had an urgent CRV, 1047 (4.8%) had a nonurgent CRV, 549 (2.5%) had a nonhemorrhagic stroke, 647 (3.0%) were hospitalized for heart failure, 901 (4.1%) had a noncoronary revascularization, 1618 (7.4%) had an incident cancer, 840 (3.8%) developed incident diabetes, 708 (3.2%) died from a vascular cause, and 960 (4.4%) died from a nonvascular cause.

### QoL and Hospital Episode Costs in the Study

**Figure 1:**
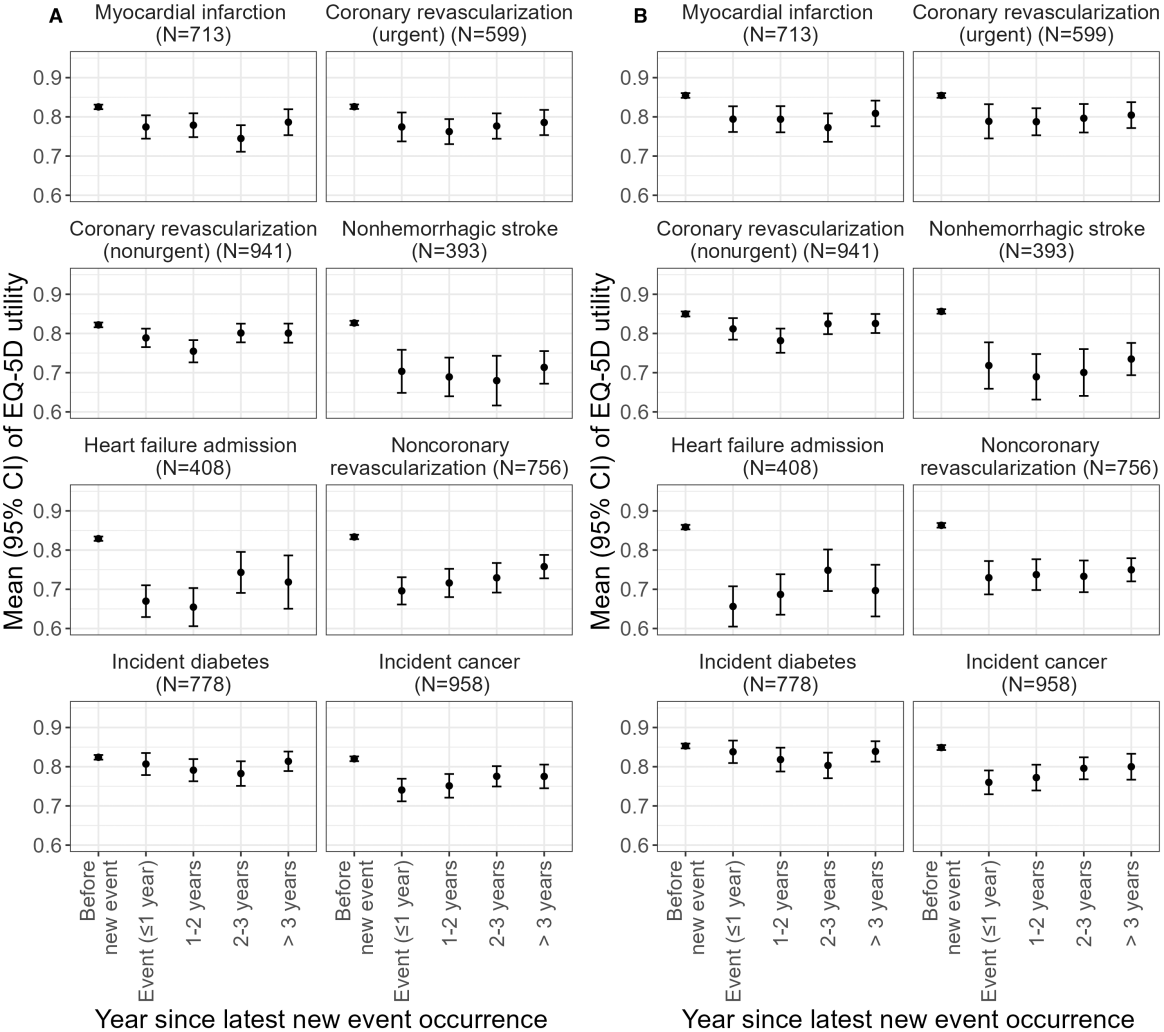
Quality of life utility of participants by category of new adverse event and time since event occurrence. UK (**A**) and US (**B**) perspectives. EQ‐5D is a standardized instrument used to measure health‐related QoL across 5 domains (mobility, self‐care, usual activities, pain/discomfort, anxiety/depression). By combining the scores of each dimension, the EQ‐5D generates a health state profile, which can be converted into a single summary QoL utility value. The QoL utility represents an individual's overall health status on a scale where 1.0 denotes perfect health, 0.0 represents death, and negative values indicate health states considered worse than death. UK or US EQ‐5D QoL utility tariffs were used, respectively. Each participant completed the EQ‐5D questionnaire at baseline and at the end of the trial. The numbers in parentheses indicate the total number of participants who experienced the particular adverse events before EQ‐5D questionnaire completion at the end of the trial. The figure presents QoL for participants who had experienced new relevant events during the trial. QoL utility before new event uses EQ‐5D questionnaires completed at entry into the study and QoL utilities within a year of event and in later years based on EQ‐5D questionnaires completed at end of trial. Participants contributed to categories of *Years since new event occurrence*, depending on the number of years from their latest relevant event to EQ‐5D measurement at end of study. EQ‐5D indicates EuroQol‐5 Dimensions; QoL, quality of life; UK, United Kingdom; and US, United States.

The QoL analysis included 19 321 participants (88.5%) who completed both the baseline and final follow‐up EQ‐5D‐5L questionnaires. At the final study follow‐up, the mean QoL utility was 0.81 (SD 0.20) (UK perspective) and 0.83 (SD 0.21) (US perspective), with 6312 of the 19 321 participants (32.7%) reporting full health (ie, no problems in each of the 5 EQ‐5D dimensions). Worse QoL was observed in the year of event and in the following years for most events (Figure 1).

**Figure 2:**
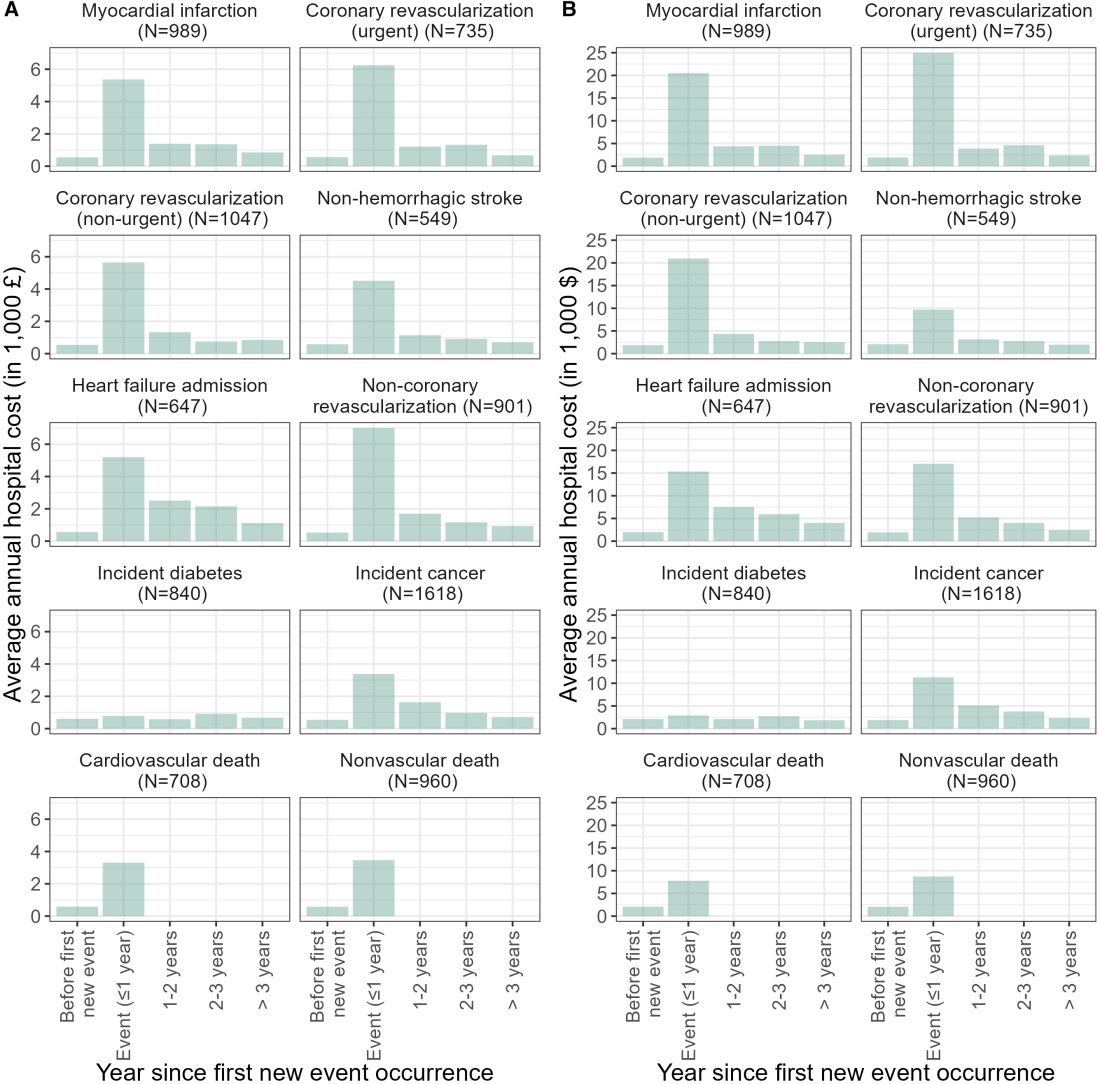
Mean annual hospital costs before, in year of, and in subsequent years of first new adverse event occurrence. UK (**A**) and US (Medicare) (**B**) health care perspectives. UK hospital care costed using Healthcare Resource Group reference costs. US hospital care costed using Medicare diagnosis‐related group costs plus professional fees. The numbers of participants who experienced the adverse events during study follow‐up are presented in parentheses. Participants contributed years of cost data to categories of *Years since new event occurrence*, depending on time since their first relevant event. Their annual cost data in years before event occurrence contributed to the *Before first new event* category. All hospital costs at 2019 prices. UK indicates United Kingdom; and US, United States.

All 21 820 participants contributed to the cost analyses. The average annual hospital costs across all participants were £607 (SD £2402) and $2114 (SD $6188) from the UK and US health care systems perspectives, respectively. Hospital costs were reported in 20% of the 96 962 person‐years of follow‐up during the study. Hospital costs peaked in the year of event occurrence, and, except for incident diabetes, remained significantly elevated in subsequent years following nonfatal adverse events as compared with patients without a history of respective events (Figure 2).

### QoL Regression Models and Impacts of Events on QoL

**Figure 3:**
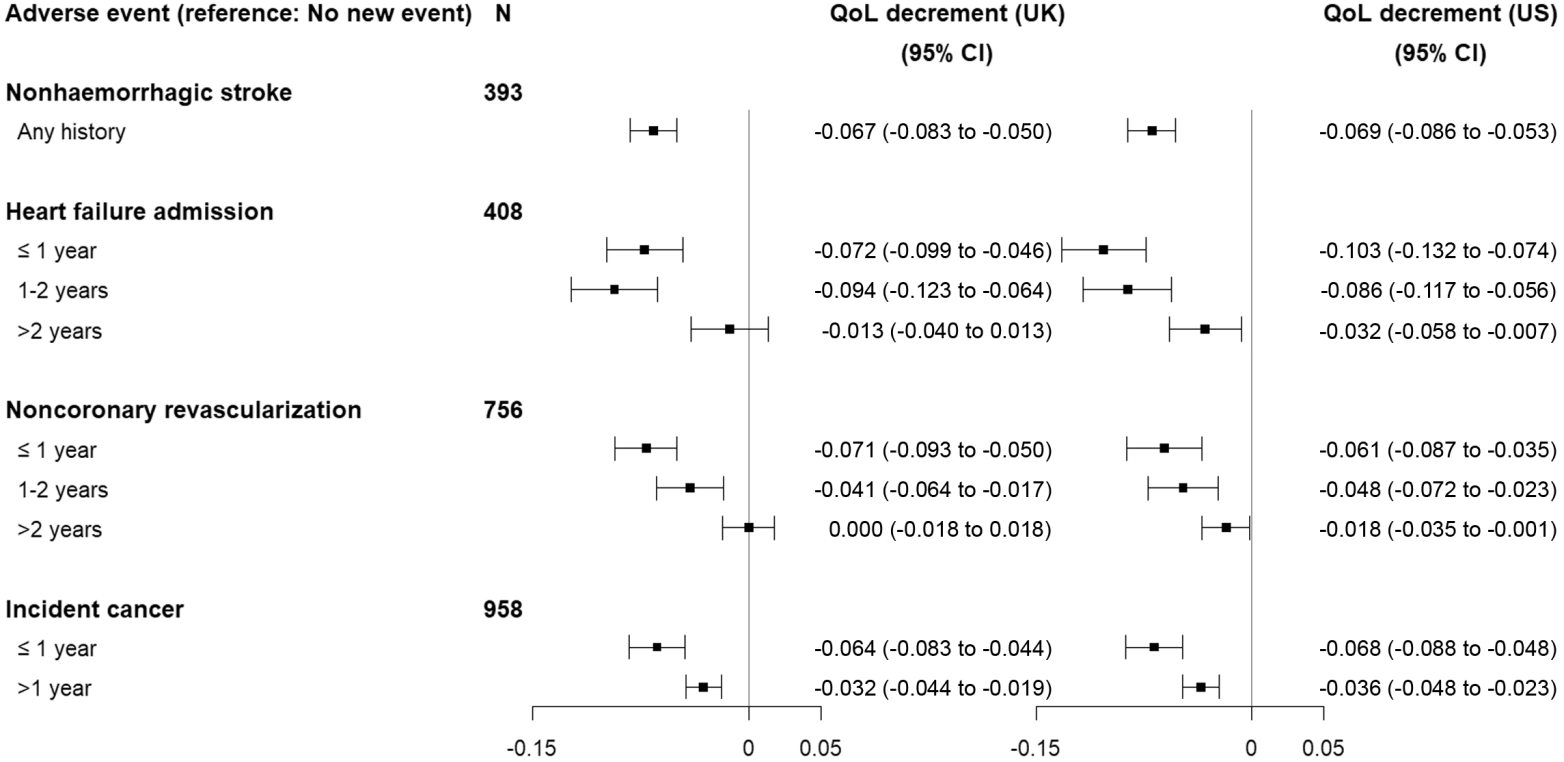
Quality of life (QoL) reductions associated with new adverse events in secondary prevention of CVD, UK and US perspectives. Data of the 19 321 REVEAL participants randomized in Europe and North America was used in both analyses from the UK and US perspectives. EQ‐5D utility values for all 19 321 contributing participants were calculated using the UK or US EQ‐5D value sets, respectively. EQ‐5D is a standardized instrument used to measure health‐related QoL across 5 domains (mobility, self‐care, usual activities, pain/discomfort, anxiety/depression). By combining the scores of each dimension, the EQ‐5D generates a health state profile, which can be converted into a single summary index score. The index score represents an individual's overall health status on a scale where 1.0 denotes perfect health, 0.0 represents death, and negative values indicate health states considered worse than death. QoL reductions associated with adverse events, by duration of time between latest adverse event from each category and EQ‐5D QoL measure at the end of the trial (≤1 year, 1–2 years, and >1 or >2 years), were estimated using a linear regression model with adjustments for sociodemographic and clinical characteristics. We were unable to detect QoL reductions associated with myocardial infarction, coronary revascularization (urgent, nonurgent), and incident diabetes, so these events were not included in the model. CVD indicates cardiovascular disease; EQ‐5D, EuroQol‐5 Dimensions; N, number of people who experienced the event in the study before QoL measure at final follow‐up in the study; QoL, quality of life; REVEAL, Randomized Evaluation of the Effects of Anacetrapib Through Lipid Modification; UK, United Kingdom; and US, United States.

The linear regression models of QoL at final follow‐up for the UK and US health care perspectives respectively, are presented in Figure 3 and in Table S1. The mean EQ‐5D utility for the reference patient: a man aged 67 years old, with previous MI history who never drank or smoked, had systolic blood pressure of <125 mm Hg, body mass index of <25 kg/m^2^, glomerular filtration rate of ≥60 mL/min per 1.73 m^2^, urine albumin‐to‐creatinine ratio of <3 mg/mmol, and no adverse event since entering trial was 0.929 (SE 0.011) in the United Kingdom and 0.967 (SE 0.011) in the US setting. In both settings, the highest QoL reduction in the year of event occurrence was associated with heart failure admission (United Kingdom: −0.072 [95% CI, −0.099 to −0.046], United States: −0.103 [95% CI, −0.132 to −0.074]), followed by noncoronary revascularization (United Kingdom: −0.071 [95% CI, −0.093 to −0.050], United States: −0.061 [95% CI, −0.087 to −0.035]), nonhemorrhagic stroke (United Kingdom: −0.067 [95% CI, −0.083 to −0.050], United States: −0.069 [95% CI, −0.086 to −0.053]), and incident cancer (United Kingdom: −0.064 [95% CI, −0.083 to −0.044], United States: −0.068 [95% CI, −0.088 to −0.048]).

For nonhemorrhagic stroke, this impact on QoL persisted in all subsequent years. For other adverse events, QoL improved in subsequent years though for most remained lower compared with not experiencing new event. Reductions in QoL were observed in years following heart failure admission (United Kingdom: 1–2 years after year of event: −0.094 [95% CI, −0.123 to −0.064], >2 years: −0.013 [95% CI, −0.040 to 0.013]; United States: 1–2 years after year of event: −0.086 [95% CI, −0.117 to −0.056]; >2 years: −0.032 [95% CI, −0.058 to −0.007]), noncoronary revascularization (United Kingdom: 1–2 years after year of event: −0.041 [95% CI, −0.064 to −0.017], >2 years: 0.000 [95% CI, −0.018 to 0.018]; United States:1–2 years after year of event: −0.048 [95% CI, −0.072 to −0.023]; >2 years: −0.018 [95% CI, −0.035 to −0.001]) and incident cancer (for all years following the year of event United Kingdom: −0.032 [95% CI, −0.044 to −0.019]; United States: −0.036 [95% CI, −0.048 to −0.023]) (Figure 3).

We were unable to detect reductions in QoL associated with MI, CRV (urgent or nonurgent), and incident diabetes so these events were not included in the final models. Independent of the occurrence of adverse events, older age, being female, a current smoker, obese, having atrial fibrillation, and reduced kidney function were all associated with lower QoL (Table S1).

The impacts of event were similar in scenario analyses of QoL reduction using only UK or North American participant data, though with increased uncertainty due to the smaller number of events (Table S2). The estimated QoL reductions with events were slightly larger when the adjustment for QoL at baseline was excluded from the models (Table S3). In the scenario analysis of Chinese participants (excluded from the main analysis), the QoL reductions associated with nonhemorrhagic stroke and incident cancer events were more than double those for Western populations, there was a reduction in QoL associated with MI and an increase in QoL associated with an urgent CRV (Table S4).

### Hospital Care Costs Models and Impacts of Adverse Events on Hospital Care Costs

The results from the specification tests and comparison of predictive performance of different model specifications are reported in Table S5. The 2‐part GLM with identity‐link function and gamma variance outperformed the single equation models and the other 2‐part specifications and was chosen to model annual hospital costs from both the UK and US health systems perspectives (Tables S6 and S7). The additional annual hospital costs associated with key adverse events are presented in Table 2.

**Table 2:** Additional Annual Hospital Costs Associated With Selected New Adverse Events From the UK and US (Medicare) Health Care Perspectives

Adverse event	Additional UK hospital cost (95% CI), UK£	Additional US (Medicare) hospital cost (95% CI), US$
Nonfatal MI with CRV (urgent)* (n=482)		
≤1 y	5614 (5179 to 6089)	24 722 (23 800 to 25 764)
>1 y	515 (337 to 712)	826 (454 to 1235)
Nonfatal MI without CRV (urgent)* (n=398)		
≤1 y	2603 (2200 to 3068)	7972 (7152 to 8792)
>1 y	360 (197 to 532)	829 (457 to 1239)
CRV (urgent) without MI* (n=228)		
≤1 y	4674 (4254 to 5149)	15 251 (14 338 to 16 213)
>1 y	100 (−29 to 244)	604 (184 to 1095)
CRV (nonurgent) without MI† (n=976)		
≤1 y	4651 (4338 to 4988)	17 539 (16 931 to 18 167)
>1 y	292 (181 to 410)	1114 (812 to 1473)
Nonfatal nonhemorrhagic stroke (n=506)		
≤1 y	3049 (2569 to 3620)	5982 (5760 to 6202)
>1 y	291 (132 to 461)	657 (418 to 881)
Heart failure admission (n=647)		
≤1 y	3182 (2727 to 3670)	9898 (8926 to 10 857)
>1 y	861 (672 to 1061)	2484 (1992 to 3011)
Noncoronary revascularization (n=901)		
≤1 y	5830 (5437 to 6248)	14 133 (13 493 to 14 829)
>1 y	557 (407 to 704)	1619 (1260 to 1976)
Incident diabetes (n=840)		
Any history	76 (37 to 117)	287 (145 to 434)
Nonfatal incident cancer (n=1330)		
≤1 y	2329 (2115 to 2525)	8438 (7868 to 9092)
1–2 y	645 (530 to 768)	2263 (1890 to 2690)
>2 y	478 (377 to 585)	1657 (1294 to 2041)
Fatal MI (n=109)	98 (−302 to 528)	1747 (858 to 2931)
Fatal nonhemorrhagic stroke (n=43)	2023 (1086 to 3256)	3771 (1851 to 6485)
Vascular death without MI and without nonhemorrhagic stroke‡ (n=497)	807 (493 to 1170)	1840 (1501 to 2187)
Fatal incident cancer (n=288)	1464 (1138 to 1824)	5264 (4060 to 6558)
Nonvascular death without incident cancer‡ (n=422)	1429 (1202 to 1693)	2505 (2173 to 2833)

Data of 21 820 REVEAL study participants recruited in Europe and North America contributed to this analysis of hospital inpatient care costs. Hospital admissions were costed using UK National Health Service or US Medicare program reference costs, respectively. All hospital costs at 2019 prices. Events referred to *fatal* if followed by death in the respective annual period, otherwise *nonfatal*. CRV indicates coronary revascularization procedure; MI, myocardial infarction; REVEAL, Randomized Evaluation of the Effects of Anacetrapib Through Lipid Modification UK, United Kingdom; and US, United States.

* Statistically significant (*P*<0.01) interaction between MI and CRV (urgent) occurring in same year was observed for US costs only.

^†^ Statistically significant (*P*<0.01) interaction between MI and CRV (nonurgent) occurring in same year was observed for US costs only.

^‡^ Other interactions (MI and vascular death, nonhemorrhagic stroke and vascular death, and incident cancer and nonvascular death) were observed for both UK and US annual hospital costs.

In the United Kingdom, the highest additional annual hospital costs were in the year with noncoronary revascularization £5830 [95% CI, 5437 to 6248], nonfatal MI with urgent CRV £5614 [95% CI, 5179 to 6089], urgent CRV without MI £4674 [95% CI, 4254 to 5149], and nonurgent CRV without MI £4651 [95% CI, 4338 to 4988]. Additional hospital costs were also observed in the year with heart failure admission, nonhemorrhagic stroke, MI without CRV, incident cancer, nonhemorrhagic stroke, and nonvascular or vascular death (Table 2). In the United States, the events associated with the highest additional annual hospital costs (Medicare) in year of event were nonfatal MI with urgent CRV $24 722 (95% CI, 23800 to 25 764), nonurgent CRV without MI $17 539 (95% CI, 16 931 to 18 167), urgent CRV without MI $15 251 (95% CI, 14 338 to 16 213), and noncoronary revascularization $14 133 (95% CI, 13 493 to 14 829). Additional hospital costs were also observed in the year with heart failure admission, incident cancer, MI without urgent CRV, nonhemorrhagic stroke, incident cancer, and nonvascular or vascular death (Table 2).

Most of the adverse events were also associated with additional long‐term annual hospital costs, with admission for heart failure, noncoronary revascularization, and incident cancer associated with the largest long‐term impacts from both the US and UK perspectives (Table 2). Significant interactions were observed in both the United Kingdom and United States (Medicare) analyses for co‐occurrences in the same year of (1) MI or nonhemorrhagic stroke and vascular death and (2) incident cancer and nonvascular death. Two further significant interactions were observed in the United States (Medicare) analysis for the co‐occurrence of MI and CRV (urgent or nonurgent) (Tables S6 and S7).

Results were similar in scenario analyses that used only UK and only North American participants when reporting additional annual hospital costs associated with events, although uncertainty increased due to smaller number of contributing events (Table S8).

An Excel program for the implementation of the health‐related QoL and hospital care cost models accompanies the article (Data S3).

## Discussion

In this study, a number of key vascular and nonvascular events experienced by patients with a history of CVD were found to have important and long‐lasting impacts on QoL and hospital costs. These impacts were derived using high‐quality data from a large study with adjudicated event end points and about 4 years of follow‐up. In both UK and US health care system perspectives, patients experiencing stroke, heart failure, noncoronary revascularization, and incident cancer incurred the largest QoL reductions, whereas CRV and noncoronary revascularization procedures were associated with the largest additional hospital costs. Although the QoL reductions associated with adverse events were largely comparable, it was considerably more expensive to receive treatment in the United States.

Study data from Europe and North America were used, together with respective UK or US QoL utility weights and unit costs, to assess the impacts of adverse events on QoL and hospital inpatient costs from the UK and US perspectives, respectively. Only about 20% to 40% (United Kingdom) or 30% to 45% (North America) of key disease events occurred among participants recruited in the United Kingdom or North America, respectively, and analyses using these smaller numbers of events produced less precise estimates as indicated by sensitivity analyses. Furthermore, there were no important differences in likelihood of inpatient admission and, following use of the same QoL utility weights and unit costs, nor were there differences in the QoL utility and hospital inpatient costs associated with key disease events between participants recruited in the United Kingdom, North America, and the rest of Europe. We have also reported in sensitivity analyses separate estimates derived using only participants recruited in United Kingdom and only participants recruited in North America, with estimates in line with the main study findings. Moreover, the study assessed the impact of events on all subsequent inpatient hospital costs, cardiovascular and nonvascular, in recognition of the increased risk, not only of recurrent cardiovascular events and other cardiovascular conditions but also of cognitive decline,^31^ diabetes,^32^ kidney,^33^ and other conditions.

QoL reduction associated with cardiovascular adverse events in predominantly secondary CVD prevention populations have been previously reported from the perspectives of UK^3^, ^23^ and US^3^, ^34^ health care (Table S9). As with the present study, previous studies also used adverse event and QoL data collected from large randomized controlled trials. Only 1 of those studies reported QoL reductions due to adverse events from the perspectives of both UK and US population valuations of QoL^3^ and, similar to our present study, estimated reductions were very similar between the countries. Smaller QoL reductions are reported in the present and another recent study^34^ compared with earlier studies. In the present study, we did not detect changes in QoL following MI, nor following CRV procedures. Similarly, studies in people with diabetes have reported that impacts of cardiovascular events on QoL may have decreased over time.^35^ This suggests that new estimates of impacts of disease events on QoL in contemporary clinical practice are required to better inform policy decisions. Stroke, heart failure admission, and noncoronary revascularization, however, remain the adverse events with the highest detrimental effects on QoL in secondary CVD populations. Our study is the first to assess the QoL reductions related to incident cancer and incident diabetes, established concomitant disease hazards. Although we found incident cancer was associated with a long‐lasting reduction in QoL, we did not find incident diabetes to be associated with reductions in QoL, but this may be due to the short duration of follow up in our study.

We report estimates of hospital care costs associated with new cardiovascular events in patients with a history of CVD from both UK and US public health care perspectives. Although the additional costs were highest in the year of event, costs were also increased in subsequent years, and analyses that fail to accurately incorporate these long‐term costs may produce serious underestimates of the health care costs of these events and the savings from averting them. The comparability of estimated costs with previous work is limited by different patient populations and costing methodologies. Nevertheless, a similar ordering of costs by event types have been reported.^13^, ^23^, ^36^ Hospital costs in the United States were, on average, 2.5 times higher for a typical patient in the United States compared with the United Kingdom. Higher US hospital costs were also reported for MI, stroke, heart failure, and CRV in previous studies.^13^, ^36^ Our study is, however, the first to assess the long‐term trajectory of these additional costs for a large set of vascular and nonvascular events.

Our study has a number of limitations. First, we focused on a number of major vascular events targeted in secondary cardiovascular prevention and therefore of interest in policy analyses of new cardiovascular therapies. Second, the study is based on clinical trials data and there may be volunteer bias whereby healthier individuals enrolled in the study. This is a common limitation that needs to be balanced against the high quality of data collected in clinical trials, which can be used for analyses such as these. Nevertheless, efforts to collect sufficiently detailed data in the real world should be made. Third, study participants who died (1668 [7.6%]) or did not provide a QoL measure at the end of the study (831 [3.8%]) did not contribute to the QoL modeling so there may be bias. In applications, QoL is used concurrently with survival in assessing the net effects of treatments and our analysis is in line with how these estimates are intended for use. This does make the assumption that the estimated QoL reductions apply up to the time before death in those participants who died. This may not be true if, for example, participants who survived have experienced better recovery from adverse events and thus may have better QoL than participants who experienced an event and later died before QoL measurement. Fourth, hospital costs included in the present study likely underestimate the total hospital care cost for 2 reasons. No data were available in the study about patients' use of hospital outpatient services and therefore these costs were not included. Furthermore, US Medicare and UK National Health Service costs were used, which are lower than private health care costs. Therefore, the costs presented reflect only the respective country's public health care admissions and costs. Finally, we also did not assess the primary and ambulatory care costs associated with adverse events. Previous CVD burden studies have indicated that the inpatient hospital care costs account for the majority of health care costs of coronary and cerebrovascular diseases (57% and 79%, respectively in 2015, United Kingdom^37^; 53% and 55%, respectively in 2016, United States^38^). Moreover, previous studies have indicated that the share of hospital costs is substantially larger in the year of the cardiovascular event when the excess costs are highest (93%–96% versus 65%–75% in subsequent years^39^ in the United Kingdom; >65% versus about 40% in subsequent years, excluding physician fees, in Canada^40^). The present study included physician fees for care during hospital admissions, ensuring comprehensive assessment of hospital admission cost and therefore the majority of the excess cost.

Despite these limitations, our study benefits from high‐quality detailed data of a large trial cohort that allowed us to report that the occurrence of key vascular and nonvascular events in patients with well‐managed CVD continues to affect significantly individuals' QoL and hospital care resources.

## Conclusions

In conclusion, the impacts of vascular and nonvascular events on QoL and hospital costs reported in this study could inform assessments of net effects and cost‐effectiveness of further CVD therapies to reduce CVD risk in people with history of CVD. To assist such applications, an Excel program for the implementation of the health‐related QoL and hospital care cost models is available with the article (Data S3).

## Sources of Funding

This work was supported by grants from Merck & Co., Inc. to the University of Oxford. Support was also provided by the British Heart Foundation (including direct support for Professor Hopewell through grant FS/14/55/30806), the UK Medical Research Council (which funds the Medical Research Council Population Health Research Unit in a strategic partnership with the University of Oxford), the UK National Institute for Health Research Clinical Research Network, Health Data Research UK, the National Institute for Health Research Oxford Biomedical Research Centre, and the National Institute for Health Research Barts Biomedical Research Centre (NIHR203330). The REVEAL trial was designed, conducted, analyzed, and interpreted by independent investigators in the Clinical Trial Service Unit at the University of Oxford (the regulatory trial sponsor), Oxford, UK in collaboration with the Thrombolysis in Myocardial Infarction Study Group at Brigham and Women's Hospital and Harvard Medical School in Boston, MA, along with other members of the Steering Committee and Merck & Co, Inc., NJ. Merck funded the trial and provided trial drugs. The coauthors prepared the article, which was reviewed and approved by the trial Steering Committee. The decision to submit it for publication was independent of all funding sources. For the purpose of Open Access, the author has applied a CC BY public copyright license to any Author Accepted Manuscript version arising from this submission.

## Disclosures

Drs Lui, Williams, Keng, Sammons, and Chen; Professors Hopewell, Gray, Bowman, and Mihaylova; and Professor Sir Landray are employed by the Nuffield Department of Population Health, University of Oxford. The Nuffield Department of Population Health has a staff policy of not taking any personal payments directly or indirectly from industry (with reimbursement sought only for the costs of travel and accommodation to attend scientific meetings; see https://www.ndph.ox.ac.uk/files/about/ndphindependenceofresearchpolicysep21.pdf).

## Supporting information

Data S1–S3
Tables S1–S9
References 41–44
