# Supplementary material for: Impact of New Cardiovascular Events on Quality of Life and Hospital Costs in People With Cardiovascular Disease in the United Kingdom and United States
Source: J Am Heart Assoc. 2023 Sep 26;12(19):e030766. doi: 10.1161/JAHA.123.030766 (PMC7615160; doi:10.1161/JAHA.123.030766)
Supplement: Supplementary file 1 — Data S1–S3 Tables S1–S9 References 41–44 [file JAH3-12-e030766-s001.zip › jah38831-sup-0001-Data S1, S2 & Tables S1 to S9.pdf]

Supplemental Material

Data S1. REVEAL Collaborative Group

Steering Committee

MJ Landray, L Bowman (Principal Investigators); R Collins (Chair); E Braunwald (Deputy Chair); JC Hopewell (Trial Statistician); L Jiang, CP Cannon, SD Wiviott, J Armitage, R Haynes, AP Maggioni, CE Angermann, G Ertl, C Wanner, T Pedersen, S Goto, T Teramoto (Regional Representatives); C Baigent, P Barter, Y Chen, Z Chen, A Gray, B Mihaylova, P Sleight, J Tobert (other voting members); R Blaustein, P DeLucca, Y Mitchel, G van Leijenhorst (non-voting Merck representatives)

Data Monitoring Committee

P Sandercock (Chair), D DeMets, J Kjekshus, J Neuberger, A Tonkin; J Emberson[+] ([+]non-voting DMC Statistician)

Lipid Monitoring Committee

C Granger (Chair), H Colhoun; K Wallendszus (non-voting statistical programmer)

Coordinating Centres

Central Coordinating Office and UK (Clinical Trial Service Unit, University of Oxford): *Management Committee:* MJ Landray, L Bowman (principal investigators), J Barton, C Bray, R Dayanandan, C Knott, M Lay, K Murphy, E Wincott; *Administration and support:* J Barton, C Bray, R Dayanandan, K Murphy, E Wincott (coordinators), P Achiri, S Barry, S Bateman, A Brewster, S Briggs, R Brown, A Burke, E Butler, L Cobb, A Collet, J Crowther, L Cureton, S Danesh-Pour, S Fathers, L Fletcher, K Frederick, T Gordon, M Gray, J Heineman, S Howard, D Jackson, N Lam, R Lee, O Machin, Z Madgwick, M Matthewson, J Nolan, M Nunn, A Panicker, L Pank, E Pearson-Burton, S Pickworth, Y Qiao, A Radley, K Roby, J Sayer, S Shah, K Taylor, H Thorne, A Timadjer, K Vandenberg, M Wickman, M Willett, J Woods, H Yu; *Clinical support and adjudication:* J Armitage, T Aung, L Bowman, R Bulbulia, F Chen, R Clarke, R Haynes, W Herrington, P Judge, MJ Landray, D Lewis, R Llewellyn-Bennett, M Mafham, D Preiss, C Reith, E Sammons, B Storey, J Tomson, E Waters; *Computing & validation:* A Baxter, M Lay, R Goodenough (coordinators); R Ait-Sadi, M Arnold, I Barton, C Berry, G Blower, J Booth, E Brown, Y Bu, P Cleverley, G Coates, J Cox, M Craig, G Cui, P Dalton, L Danel, C Daniels, C Dawe, A Field, S Gilbert, P Harding, K Jayne, R Kurien, G Lancaster, A Maskill, A McDougall, Y Mostefai, S Mulay, A Munday, A Murawska, N Prajapati, S Ramesh, R Reid, S Syed, H Todd, A Young, A Young, W Zhu; *Statistical analysis:* JC Hopewell (study statistician), K Wallendszus (statistical programming coordinator), M Arnold, S Parish, W Stevens, E Valdes-Marquez; *NDPH Wolfson Laboratory:* M Hill (laboratory director), S Clark, K Emmens, G McClean, M Radley, J Wintour (laboratory coordinators); M Allworth, A-M Beneat, C Bird, L Boggs, A Casey, T Chavagnon, K Chung, R Chung, L Cockram, R Cox, J Douglas, L Finnegan, H French, N Goodwin, A Gordon, J Gordon, C Guest, S Hazim, J Hill, R Hrusecka, M Lacey, N Luker, S Mulligan, ME Obrero, N Plunkett, L Sansom, R Shellard, J Taylor, P Taylor, J Tyler, L Weaving, J Wheeler, T Williams, M Yeung; *Monitors and trainers:* C Knott (coordinator); S Beebe, K Bowsher-Brown, J Dabrowski, J Henderson, J James, H Lochhead, V Toghill, L Wright, L Young;

China (China-Oxford Centre for International Health Research, Fuwai Hospital, Beijing): *Regional coordinator:* L Jiang; *Clinical support and adjudication:* W Hundei, J Liu, J Qu, H Zhang; *Administration and support:* H Dai, F Feng, L Hou, J Li, L Ma, S Niu, R Tang, S Wang, X Wei, M Xie, X Yan, M Yang, Y Zhang, L Zhang, A Zhang, S Zhang, L Zhao, H Zhong; *Monitors and Trainers:* L Chen, Y Gao, L Li, H Yang; *IT Support:* J Zhang;

Germany (Comprehensive Heart Failure Center [CHFC] and Department of Internal Medicine I, University Hospital, Würzburg):

Regional coordinators: CE Angermann, G Ertl, C Wanner; *Clinical support and adjudication:* S Brenner, M Heldmann, B Kraus, B Meyer; *Administration and support:* M Fajardo-Moser, C Hartner; *Monitors and Trainers:* A Knoppe, D Pop-Marschall, J Renner, U Saemann;

Italy (Centro Studi Associazione Nazionale Medici Cardiologi Ospedalieri [ANMCO], Florence):
Regional coordinator: AP Maggioni; *Clinical support and adjudication:* G Fabbri; *Administration and support:* A Lorimer, D Lucci, B Bartolomei Mecatti; *Monitors and Trainers:* M Ceseri, E Baldini, S Benoni, F Bianchini, P Ferruzzi, M Miccoli, S Musio, F Ramani; *IT Support:* M Gorini, G Orsini;

Japan (School of Medicine Multiregional Study Office, Tokai University):
Regional coordinators: S Goto, T Teramoto; *Clinical support and adjudication:* E Kato, K Tawara, A Tomita; *Administration and support:* S Kitamura, Y Saitoh, M Shimizu, S Shiozaki, K Soeda, A Tanaka; *Monitors and Trainers:* E Kato; K Tawara; A Tomita;
[Note: These collaborators participated in preparations to initiate recruitment in Japan, but it was subsequently planned not to extend recruitment there.]

North America (TIMI Study Group, Boston):
Regional coordinators: E Braunwald, CP Cannon, SD Wiviott; *Clinical support and adjudication:* A Eisen, E Kato, D Steen; *Administration and support:* P Fish (director of operations), S MacDonnell, J Kent, A McCagg (lead coordinators), E Greene, D Klements, K Washington; *Monitoring and training (Covance, Princeton):* A Davis, M Goeres, J Joyce, J Koen (lead monitors), J Colicchia, J Domercant, V Foster, C Fox, C Gennusa, R Hollis, Y Kassa, A Kelley, V Magloire, C Owens, N Yeh;

Scandinavia (MSD, Copenhagen):
Regional coordinator: T Pedersen (*Ullevål Universitetssykehus HF, Oslo*); *Coordinators:* K Arnesson, S Mosegaard; *Clinical support and adjudication:* K Andersen, S Haywood, A Osmanagic, C Pilgaard Madsen, E Rebnord, K Serup-Hansen, M Tarras Wahlberg; *Administration and support:* Denmark: K Hannibal, T Johansen, L Rasmussen, A Sloth; Finland: P Kiuru, M Lauronen, A-S Leinonen, T Mononen, M Vuola, S Wiik; Norway: H Hovdal, C Lien, S Svingen, P Singh, I Thorsby, E Westerheim; Sweden: P Bergsten, L Bergvall, H Castedal, A-C Cederholm, L Froberg, A Johansson, L Jonsson, P Martin, T Rasmusson, S Wiik-Karu; *Monitoring and training:* Denmark: H Diget, O Moll, S Snejbjerg, G Sørensen; Finland: S Eronen, S Roine, T Vaine; Norway: V Bjørhovde, L Edvardsen, S Saether; Sweden: Å Blechert, I Ek, L Hedlöf, J Levin, D Vlaheli;

Local Clinical Centres

Canada
National coordinator: J Genest; *Collaborators: Brampton Research Associates, Brampton, ON:* M Gupta, A Burgess, C Dela Cruz, S Harnden, S Hirjikaka, E Mallari, Y Thevakumaran; *Cambridge Cardiac Care Inc., Cambridge, ON:* A Pandey, J Lake, M Pandey, C Wang; *Centre de Dépistage et Recherche Cardiovasculaire Rive-Sud, Longueuil, QC:* E Sabbah, I Chausse, F Deslongchamp, J Lavoie; *Centre de Recherche Medialpha, St. Lachine, QC:* G Sabe-Affaki, S Fontaine; *Centre intégré de santé et de services sociaux de Lanaudière - Hôpital Pierre-Le Gardeur, Terrebonne, QC:* G Gosselin, M David, K Drouin, N Lachance, C Masson, M Pashko, C Tremblay; *Clinique Sante Cardio MC, Montreal, QC:* C Constance, M Gauthier; *CSSSNL/CHRDL, Saint-Charles-Borromee, QC:* S Kouz, C Fleury, V Lemay-Chretien, N Roberge, M Roy; *Discovery Clinical Services LTD, Victoria, BC:* G Hoag, R Standring, L Warke; *Dr. Stephen Pearce, Inc, Surrey, BC:* S Pearce, L Breakwell, T Cleveland, D Kastanis; *Heart Care Research, Oshawa, ON:* R Bhargava, C Stafford, C Stata; *Heritage Medical Research Clinic, Calgary, AB:* T Anderson, D Brown, B Madden, M Pajevic, D Ramadan, B Smith; *James Cha MD, Oshawa, ON:* J Cha, J Otis; *Newmarket Cardiology Research Group, Newmarket, ON:* R Zadra, A Harwood, C McPherson, C Rackham; *Oshawa Clinic, Oshawa, ON:* A Bakbak, S Baghiana, K Gibney, L Swailes; *Q&T Research Outaouais Incorporated, Gatineau, QC:* P Nault, K Audet, C Roy, E St-Amour, I Tremblay; *Viacar Recherche Clinique Inc., Greenfield Park, QC:* R Chehayeb, C Lepage; Vizel Cardiac Research, Cambridge, ON: S Vizel, B Fox;

China

National coordinator: L Jiang; *Collaborators: Affiliated Zhongshan Hospital of Dalian University, Department of Cardiology:* Q Yu, L Chi, F Liu; *Baotou Central Hospital, Department of Cardiology:* R Zhao, X Li, Y Qian, J Wang; *Beijing Chao-Yang Hospital, Capital Medical University, Department of Cardiology:* X Yang, M Chen, X Lin, F Zhang; *Beijing Liangxiang Hospital of Fangshan District, Department of Cardiology:* X Fang, Q Yu, W Su, X Zhu; *Changsha Central Hospital, Department of Cardiology:* H Dai, L Huang, G Ye, Y Zhao; *China-Japan Union Hospital of Jilin University, Department of Cardiology:* P Yang, M He, B Li; *Dalian Municipal Central Hospital, Division of Cardiology:* Y Zhi, L Sun, L Xiao, Y Yuan; *Fenyang Hospital of Shanxi Province, Department of Cardiology:* R Guo, Q Wang, Y Wang; *Fuwai Hospital, 25th Ward:* J Li, Y Gao, Y Guo, L Li, M Zhai, L Zhang; *Fuwai Hospital, Department of Cardiology:* Y Yang, J Song; *Fuwai Hospital, Department of Heart Surgery:* Z Zheng, X Wang, Y Zhao, C Zhao; *Houma City People Hospital, Department of Cardiology:* Z Wang, C Li; *Hulun Buir People's Hospital, Department of Geriatrics:* Z Cui, X Zhang, L Zhao*; Inner Mongolia Autonomous Region of Traditional Chinese-Mongolian Medicine Hospital, ICU:* H Su, X Huang, R Zhang; *Inner Mongolia Baogang Hospital, Department of Cardiology:* Z Ge, D Liu, Q Liu; *Inner Mongolia People's Hospital, Department of Cardiology:* Y Han, W He, Y Zhang; *Jilin City Central Hospital, Department of Cardiology:* D Qian, L Liu, X Yao; *Jilin Province People's Hospital, Department of Endocrinology:* Y Du, L Song; *Liaoning Shenyang Sujiatun District Central Hospital, Department of Cardiology:* H Che, D Li, C Sun; *Peking Union Medical College Hospital, Department of Cardiology:* S Zhang, H Bai, W Chen, Y Han, Z Liu, J Yang; *Peking University Third Hospital, Department of Cardiology:* Z Li, J Bai, F Wang; *Qingdao Municipal Hospital, Department of Cardiology:* X Wang, C Xing, Y Yao; *Qingdao Fuwai Hospital, Chinese Academy of Medical Science, Department of Cardiology:* X Jiang, Y Yang, Y Dong, G Wu, B Zhang; *Shanghai Changzheng Hospital, Department of Cardiology:* Z Wu, W Chen, Y Chu, X Gu; *Shanxi Cardiovascular Hospital, Department of Cardiology:* B Li, J Wang, X Chai, H Zhang; *Shanxi Provincial People's Hospital, Department of Cardiology:* H Zhang, S Sun, L Tong; *Shengjing Hospital of China Medical University, Department of Cardiology:* X Li, S Ma, H Li, J Liu, X Liu, J Shi; *Shuangshan Hospital of Anshan, Department of Cardiology:* R Xiao, X Li, R Wu; *Suzhou Kowloon Hospital, Shanghai Jiaotong University Medical School, Department of Cardiology:* F Liu, X Meng, B Shao, T Zhang; *Tai Yuan City Centre Hospital, Department of Cardiology:* X Chen, T Feng, L Huo, X Shang; *The Affiliated Hospital of Medical College Qingdao University, Department of Emergency Cardiology:* C Zhou, M Guo, P Li; *The Affiliated Hospital of Medical College Qingdao University, Department of Emergency Neurology:* H Pei, D Han, H Li; *The Affiliated Hospital of Xuzhou Medical College, Department of Cardiology:* D Li, C Cheng, M Huang, W Wu, T Xu; *The Affiliated People's Hospital of Inner Mongolia Medical College, Cadre Ward:* J Liu, J Xia; *The Central Hospital of Wuhan, Department of Endocrinology:* S Zhao, C Cheng, S Ding, L Guo, N Li; *The Fifth People's Hospital of Shenyang, Department of Cardiology:* Q Diao, Z Liu, H Wang; *The First Affiliated Hospital of China Medical University, Department of Cardiology:* G Qi, Z Jia, Y Meng, C Wu; *The First Affiliated Hospital of China Medical University, Department of Cardiology, Anshan Hospital:* B Liu, X Bian; *The First Affiliated Hospital of Harbin Medical University, Department of Cardiology:* W Li, M He, J Jing, B Liu; *The First Affiliated Hospital of Harbin Medical University, Department of Neurology:* L Zhang, Y Sun, X Wang, S Wu, Y Xu; *The First Affiliated Hospital of Xinxiang Medical University, Department of Cardiology:* F Lv, C Guo, J Long, Y Wang; *The First Affiliated Hospital of Zhengzhou University, Department of Cardiology:* Z Huang, X Fu, H Yao, L Zhang; *The First Hospital of Jilin University, Department of Cardiology:* Y Zheng, X Li, C Liu, Q Tong; *The First Hospital of Jilin University, Department of Endocrinology:* Q Liu, G Wang, Y Cheng, X Gang, W Guo, G He; *The First Hospital of Shanxi Medical University, Department of Cardiology:* Q Han, H Bian, L Duan, C Jin, X Wei; *The First People's Hospital of Shenyang, Department of Cardiology:* F Feng, W Xing, J Xu; *The Fourth Affiliated Hospital of China Medical University, Department of Cardiology:* Y Jin, Y Lin, X Zhou; *The Fourth People's Hospital of Shenyang, Department of Cardiology:* Y Li, X Guan, X Zhou; *The General Hospital Of AISCO, Department of Cardiology:* X Liu, H Liu, L Liu; *The General Hospital of FAW, Department of Cardiology:* H Pan, X Wang, S Zhang; *The General Hospital of Shenyang Military Command, Department of Cardiology:* Y Han, P Fan, J Li, R Ma, G Wang, P Wang; *The General Hospital of Xuzhou Mining Group, Department of Cardiology:* W Wu, L Li; *The People's Hospital of*

Liaoning Province, Department of Cardiology: Z Li, R Cui; *The Second Affiliated Hospital of Baotou Medical College, Department of Cardiology:* G Sun, F Wang, R Xie; *The Second Affiliated Hospital of Dalian Medical University, Cardiovascular Department:* P Qu, G Li, J Mei, L Wang, Q Yang, C Zhang; *The Second Affiliated Hospital of Harbin Medical University, Department of Cardiology:* B Yu, W Cao, W Du, Y Luan; *The Second Affiliated Hospital of Harbin Medical University, Department of Neurology:* W Wang, Y Zhu, H Jiao, Y Qu, Y Zhu; *The Second Hospital of Shanxi Medical University, Department of Cardiology:* Z Yang, N Du, J Li, B Liang, W Wu, H Yang; *The Second Hospital of Tianjin Medical University, Department of Neurology:* X Li, D Wang, P Zhao; *The Third People's Hospital of Dalian, Department of Cardiology:* N Li, X Liu, B Xu, D Zong; *The Third People's Hospital of Xuzhou, Department of Cardiology:* L Wang, X Tang, C Zong; *The Third Xiangya Hospital of Central South University, Department of Endocrinology:* Z Mo, P Jin, J Xiong; *Tianjin Chest Hospital, Department of Cardiology:* H Cong, X Guo, R Liang, J Zhou; *Tianjin Fourth Center Hospital, Department of Cardiology:* H Zhang, Y Liu, Z Sun; *Tianjin Medical University General Hospital, Department of Cardiology:* Y Sun, Z Wan, B Bian, Y Li, W Zhang; *Tianjin Union Medicine Centre, Department of Cardiology:* Z Yao, Y Liu, R Wang; *Tianjin Union Medicine Centre, Department of Neurology:* C Zhang, G Chen, C Ma; *Tongji Hospital, Department of Cardiology, Tongji Medical College:* D Wang, J Jiang, L Ni, H Yan; *Wuhan Asia Heart Hospital, Department of Cardiology:* X Su, J Cai, J Ma, R Zhong; *Wuhan Puai Hospital, Department of Cardiology:* Y Gu, L Hu, X Wu; *Wuxi People's Hospital, Department of Cardiology:* X Wu, Z Yang, M Chen, J Feng; *Xiangtan City Central Hospital, Department of Cardiology:* H Huang, F Ouyang, Z Sun, Y Zhou; *Xiangya Hospital Central-South University, Department of Cardiology:* T Yang, X Deng, L Peng, Y Zhao; *Xinxiang Central Hospital, Department of Cardiology:* L Liu, S Su; *Xuzhou Central Hospital, Department of Cardiology:* Q Fu, L Wang, X Zong; *Xuzhou N° 1 People's Hospital, Department of Cardiology:* H Zhang, L Li, X Liu, Y Shang; *Zhengzhou Central Hospital, Cardiovascular Medicine Department:* L Zhang, H Li, H Sun; *Zhu Zhou N° 1 Hospital, Department of Cardiology:* S Guo, Y He, L Cha, Y Lu;

Denmark

National coordinator: K Egstrup; *Collaborators: Aalborg Hospital:* E Berg Schmidt, P Dinesen, A Gammelmark, M Nielsen, T Rix, H Vadmann, K Andersen, B Christensen, L Hessing Kobbelgaard, B Mikkelsen, M Storgaard; *Esbjerg Hospital:* O Nyvad, A Rohold, K Thomsen, J Hummelshoj, A Svenningsen, L Tanggaard; *Gentofte Sygehus, Hellerup:* G Gislason, C Torp Pedersen, G von Jessen, J Larsen, J Sandberg Madsen; *Glostrup Hospital:* H Iversen, C Nielsen, J Obionu, S Simonsen, M Kjaergaard Danö, P Hornslet, T Veng-Olsen; *Herning Hospital:* O May, L Madsen, M Engbjerg Andersen, L Jensen, V Lynggaard, S Søndergaard, S Vester; *Holbæk Hospital:* N Roseva-Nielsen, V Sørensen, K Skjødeberg Christensen, M Bang Hansen, L Jensen, H Mollerup, S Voigt; *Kolding Hospital:* J Jepsen, J Gesla, L Johansen, E Zeuthen, B Bjerge Kaspersen, B Felthaus, M Løkke, L Holm Pedersen, A Schrader, L Schmidt Thomsen; *Kolesterollaboratoriet, Gentofte:* S Stender, T Brink-Kjaer, H Jonsson; *Næstved Hospital:* R Sykulski, J Thorsen, P Osterby Elin, B Stage Jensen; *Nordsjællands Hospital, Helsingor:* N Ralfkier, H Gottschalck, S Bloksgaard Nilesen; *Odense Universitets Hospital:* M Lytken Larsen, H Mickley, S Hosbond, L Saaby, M Ronn, I Rosenlund; *Rigshospitalet, Copenhagen:* P Clemmensen, P Grande, L Køber, H Andersson, S Wiberg, K Graversen, L Hedgaard, M Tarras Wahlberg; *Roskilde Hospital:* T Melchior, C Larsen, S Heinsvig, I Larsen, V Perret-Gentil; *Silkeborg Hospital:* L Frost, A Christensen, H Arp, M Mortensen, A Odgaard; *Skejby Hospital, Århus:* H Wiggers, S Poulsen, K Serup-Hansen, G Udsen; *Slagelse Hospital:* J Lomholt, H Mollerup, K Niemann; *Steno Diabetes Center, Gentofte:* M Ridderstråle, L Tarnow, T Boesgaard, T Hansen, N Safai, M Andersen, S Hansen, M Pedersen; *Svendborg Hospital:* K Egstrup, S Auscher, A Osmanagic, H Sheta, K Vinter, L Hindsgaul, M Lundgaard, L Moltrup; *Viborg Hospital:* I Klausen, B Haastrup, B Hedegaard, S Gudmundsdottir;

Finland

National coordinator: A Kesäniemi; *Collaborators: Etelä-Karjalan Keskussairaala, Lappeenranta:* E Hussi, S Valpas; *FinnMedi Oy, Tampere:* J Taurio, A Airaksinen, S Luukkonen, S Uusitalo; *Geri-Med Oy, Helsinki:* T Strandberg, E Ronkainen, C Sarti, R Tilvis, M Aaltonen, E Landström, A Punkka; *Kuopion Liikuntalääketieteen Tutkimuslaitos, Kuopio:* T Lakka, K Savonen, H Kastarinen, N

Koskinen; *Kuusankosken Terveysasema, Kuusankoski:* M-L Tuominen, A Haaraoja; *Lapin Keskussairaala, Rovaniemi:* J Laukkanen, A Hadjikov, S Hellsten, P Hiltunen; *Menoa Oy, Kinkomaa:* M Perhonen, M Valtonen, M Moilanen, M Varakas; *OYS, Oulu:* A Kesäniemi, E Eloranta, O Ukkola, P, Ojala, L Ukkola; *Pohjois-Karjala Projektisäätiö, Joensuu:* S Pihlman, M Mononen, K Hyttinen, S Lipponen; *Seinäjoen Lääkäritalo, Seinäjoki:* M Kotila, A Pöllänen, A Rajala; *Turun Yliopistollinen Sairaala, Turku:* I Kantola, T Kiviniemi, M Strandberg, J Raali, E Roine;

Germany

National Coordinator: C Wanner; *Collaborators: Ambulantes Herzzentrum, Kassel:* K-F Appel, S Appel, A Utech, P Becker, S Chmilewski, J Kuehnert, I Pietsch, A Reinemann, S Werner; *Cardiopraxis, Mainz:* G Mentz, M Drexler, I Müller-Wittlich, A Drexler, S Hobrack, K Tajouaout; *Charité Campus Virchow-Klinikum, Berlin:* H-D Düngen, T Bekfani, G Cherian, M Fritschka, L Musial-Bright, T Trippel, V Tscholl, A Baltic, S Inkrot, A Maiwald, A Pinta, M Sacirovic, Y Saewe, R Stolz; *Comprehensive Heart Failure Center [CHFC] and Department of Internal Medicine I, University Hospital, Würzburg:* CE Angermann, G Ertl, S Brenner, K Boelmans, M Breunig, F Hammer, U Hofmann, B Meyer, C Wanner, J Judex, A Knoppe, C Richter; *DRK Kliniken, Berlin:* H Voehringer, E Lianopoulos, C Opitz, M Buchholz, S Gebhardt, S Helms; *Forschungszentrum Ruhr, Witten:* T Horacek, G Kahrmann, O Stobbe, P Fink, A Günesli, J Richtstein, K Wilke; *Gefäß Zentrum Universitaetsklinik, Dresden:* N Weiss, N Jabs, A Mahlmann, S Werth, S Brilloff, M Dechert, E Festerling, M Leistner, B Sehr, I Weise; *Gemeinschaftspraxis Dr. Haggenmiller und Dr. Jeserich, Nuernberg:* M Jeserich, S Haggenmiller, S Kimmel, H-O Schoengart, M Cakir, G Eichinger, M Rupprecht; *Juedisches Krankenhaus, Berlin:* K Graf, R Thieme, E Tummos, J Ausner, L Fischer; *Kardiologie Universitaet, Magdeburg:* R Braun-Dullaeus, H Bönigk, S Meißler, A Schmeißer, H Schulz, S Uslar, D Weigt, R Gebauer, S Roeder, K Schäfer; *Kardiologische Geimeinschaftspraxis, München:* S Silber, M Basler, C Matt, P Styllou, B Bosnjak, M Huth, A Schmid, C Senger; *Kardiologische Gemeinschaftspraxis, Würzburg:* M Camerer, H Drösch, H Strömer, J Heid, R Wilsch; *Klinik am See, Ruedersdorf:* H Völler, A Jawari, S Stiehl, A Salzwedel, K Stolze; *Kerkhoff Klinik, Bad Nauheim:* V Mitrovic, L Gaede, A Peil, M Shaker; *Klinikum Bielefeld:* C Stellbrink, C Drephal, B Elberg, J Junge, E Stellbrink, T Weber, B Brettschneider, C Gruhne, M Iselt, J Kube, U Lehmann, C Potthast, S Watson; *Klinikum Coburg:* J Brachmann, M Held, C Mahnkopf, A Saleh, A Sallam, B Schertel-Gruenler, S Schnupp, U Goebel, S Rube, K Truthan; *Klinikum Frankfurt Hoechst:* C Kadel, K Lahiri, H Moellinger, M Pagitz, J Reusch, A Stadler, N Zulauf, V Anuschek, S Beißner, M Buerger, M Hagmanns, E Hickmann, C Klinger, G Rahn, J Schemann, E Tu, C Wölfl; *Klinikum Fulda:* V Schaechinger, T Pluecker, G Strupp, T Trepels, W Wahl, C Roemmelt, S Fritsch; *Klinikum Leverkusen:* P Schwimmbeck, A Fahrig, M Hautmann, A Öner, B Weidmann, I Wenzel; *Klinikum Universität München - Campus Innenstadt:* U Hofmann, M Czihal, K Hausleiter, K Kress, R Kreuzpointner, P Kuhlencordt, S Rieber, J Nuerbchen, S Roth-Zetzsche; *Klinikum Universität München - Grosshadern:* S Kääb, W Franz, C Feldmann, U Grabmaier, M Sinner, A Bongartz, C Gross, B Halter, J Sakic; *Medizinische Hochschule, Hannover:* J Bauersachs, U Bavendiek, J Pirr, K Sonnenschein, K Hohenleitner-Lührßen, A Juergens, N Schaefer; *Praxis fuer Kardiologie Dr. Bosiljanoff, München:* P Bosiljanoff, G Betzl, E Bosiljanoff, J Feger, A Kinateder; *Robert Bosch Krankenhaus, Stuttgart:* U Sechtem, S Egenrieder, A Karagianni, T Schäufele, M Voehringer, S Gruensfelder, L Hoffmann, I Wenzelburger; *SLK-Kliniken Heilbronn, Bad Friedrichshall:* T Dengler, C Loges, C Neatu, C Lindner, C Pfau; *St. Johannes Hospital, Dortmund:* H Heuer, H Bourhaial, I Dulea, B Elberg, O Guerocak, S Halberstadt, E Kemala, J Peterek, H Philips, U Dieckheuer, K Euler, B Laschewski, M Maas, J Peda; *Studienzentrum Prof. Hanefeld, Dresden:* F Schaper, E Henkel, M Teige, C Schrapel, K Waetzold; *Universität Magdeburg Lipidambulanz:* B Isermann, S Westphal, K Borucki, M Schulze, K West; *Universitäts-Herzzentrum Freiburg-Bad Krozingen:* D Trenk, W Hochholzer, S Leggewie, C Stratz, A Schiebeling-Romer, M Siefer; *Universitätsklinikum, Bonn:* N Werner, R Schueler, A Sedaghat, J-M Sinning, K Twelker, U Jones, M Lauterborn, M Lennarz, S Lubnau, A Meier, T Streuber-Bouhksas; *Universitätsklinikum, Hamburg:* S Blankenberg, M Adam, C Alternburg, M Huemmelgen, A Jagodzinski, M Karaks, K Koopmann, S Schäfer, H Schüler, K Sydow, C Thomas, E Tigges, I De Boer, M Hermes, J Nebel, C Schlesner, J Schlüter, D Sprechert, S Zbik; *Universitätsklinikum Münster Kardiologie:* J Waltenberger, D Fisscher, C Schulz;

Universitätsklinikum Münster Lipidambulanz: B Otte, L Centofante, R Kremerskothen, J Beilker, S Müller, E Schlosser; *Universitätsklinikum Regensburg:* C Birner, A Luchner, J Egresits, C Jungbauer, M Resch, P Schmid, M Buesing, C Liebl, S Sülflow; *Universitätsklinikum Schleswig-Holstein, Luebeck:* J Weil, B Brueggemann, T Graf, C Moeller, M Miodek; *Vivantes Klinikum am Urban, Berlin:* H Ince, D Andresen, A Seidel, C Sprenger, Y Stoeckicht, S Ziefle, S Forster, J Ort, S Szczesnak;

Italy

National coordinator: AP Maggioni; Collaborators: *Albano Laziale, Ospedali Riuniti Albano-Genzano:* P Midi, A Felici, F Caranzetti, M Miccoli, L Tomassini; *Aosta, Ospedale Generale Regionale-PO U.* Parini: M Sicuro, C Aillon, C Gianonatti, C Baré, P Donà; Bari, Ospedale San Paolo: P Caldarola, M Resta, M Ruggiero, M Galietti; *Bologna, Ospedale Maggiore:* G Di Pasquale, E Filippini, L Riva, S Zagnoni; *Casarano, Presidio Ospedaliero F. Ferrari:* G Piccinni, C Perrone, A Aloisi; *Cortona, Ospedale Valdichiana Santa Margherita:* F Cosmi, B Mariottoni, B Tarquini; *Cremona, Ospedale di Cremona:* S Frattini, S Pirelli, G Paradiso, S Signore; *Firenze, Ospedale San Giovanni di Dio:* C Baggiore, C Crescenti, A Leopardi, S Benoni, P Ferruzzi, P Pini; *Foggia, Ospedali Riuniti:* M Di Biase, C D'Antuono, R Ieva, I Monaco, D Montrone, S Musio; *Gubbio, Ospedale Gubbio-Gualdo Tadino:* S Mandorla, M Buccolieri, E Capponi, S Martinelli, N Piccioni, O Regni, A Iaquaniello, A Malvestiti, S Pieroni Minciaroli; *Isernia, Ospedale F. Veneziale:* C Olivieri, R Chiodi, A Masciotra; *Legnano, Ospedale Civile:* F Poletti, S De Servi, S Affinito, A Di Donato, S Messina, C Stefanin; *Lido di Camaiore, Nuovo Ospedale Versilia:* G Casolo, L Robiglio, F Vivaldi, A Buono, C Urbani; *Milano, Ospedale Niguarda:* A Alberti, E Giagnoni, T Pupilella, A Biondi, A Di Donato, A Lazzari, S Messina; *Orbassano, Ospedale San Luigi Gonzaga:* L Montagna, C Chirio, I Salvetti, M Perrelli; *Palmanova, Ospedale di Palmanova:* MG Baldin, R Cesanelli, S Boccati, G Durì; *Pavia, IRCCS -Fondazione Salvatore Maugeri:* S Priori, M Ceresa, M Zambelli, A Biondi, A Di Donato, S Messina, G Savino; *Rimini, Ospedale Infermi:* G Piovaccari, D Grosseto, P Testa, P Gaviani, A Girardi, I Manzo, G Serroni; *San Felice a Cancello, Ospedale Ave Gratia Plena:* C De Matteis, U Campidonico, C Crisci, M Falco, C Di Matteo, I Manzo; *Santa Maria Capua Vetere, Ospedale San Giuseppe e Melorio:* L Fattore, G Morello, C Nave, C Di Matteo, I Manzo; *Sarzana, Ospedale San Bartolomeo:* R Petacchi, D Bertoli, G Filorizzo, A Buono, C Urbani; *Scorrano, Ospedale Ignazio Veris Delli Ponti:* O De Donno, E De Lorenzi, L Urso, A Aloisi, A Lecci; *Sondrio, Ospedale Civile:* G Cucchi, E Gianatti; *Terni, Azienda USL Umbria 2:* G Proietti, M Bernardinangeli, G Proietti, S Serani; *Udine, AOU Santa Maria della Misericordia:* G Morocutti, T Bisceglia, C Fresco, V Andrioli, V Biundo; *Veruno, Fondazione Salvatore Maugeri:* P Giannuzzi (deceased), M Gattone, V Bolzani, M Di Ruocco, A Biondi, A Di Donato, S Messina, B Temporelli;

Japan

National coordinators: S Goto, T Teramoto; Collaborators: *Institute of Brain and Blood Vessels Mihara Hospital:* B Mihara; *National Hospital Organization Kyushu Medical Center:* Y Okada; [Note: These collaborators participated in preparations to initiate recruitment, but subsequently it was decided not to extend recruitment to Japan.]

Norway

National coordinator: O Nygard; *Collaborators: Førde sentralsjukehus, Førde:* T Vingsnes, H Sirnes, K Solheim, R Tilseth, M Vestre, A Bjorkas, I Vassbotten; *Haugesund Hjertepraksis, Haugesund:* R Rød, R Stodle; *Haukeland Universitetssykehus, Bergen:* O Nygård, C Berge, H Schartum Hansen, A Kask, K Løland, G Svingen, N Tuseth, V Vavik, E Wilberg Rebnord, B Gjellefall, S Hovland, S Nordgaard Thorsen; *MEDI3 Ålesund, Ålesund:* T Kjærnli, O Erstad, K Grödal, S Nybø, C Royset, S Stadsnes; *Nordland Hjertesenter AS, Bodø:* A Hovland, K Lappegard, J Sandvik, H Carlsen, T Enebakk, H Thunhaug; *Norsk Helseklinikk, Lierskogen:* L Solnør, P Holmstrom; *Skedsmo Medisinske Senter AS, Skedsmokorset:* K Risberg, H Hansen; *St. Olavs Hospital, Trondheim:* B Kulseng, K Lauglo, H Tevik Bjøru, T Langeng, S Salater; *Stavanger Helseforskning AS, Stavanger:* P Scott Munk, E Singsaas, A-G Larsen, S Moen, J Nilsen; *Sykehuset Innlandet HF, Hamar:* K Andersen, T Larsen, E Turkerud Söby; *Sykehuset Innlandet HF, Kongsvinger:* J Sparby, E Werenskjold; *Sykehuset Innlandet HF, Lillehammer:* M Grundtvig, M German, G Szacinski; *Sykehuset Telemark, Skien:* J Hysing, J Thalamus, E Flagstad, H Rosland; *Ullevål*

Universitetssykehus HF, Oslo; T Pedersen, T Klemsdal, L Bergengen, R Kleve; *UNN Hjerte/Kar Poliklinikk, Tromsö:* A Skogsholm, K Larsby, I Holde, R Jonassen, M Nilsen; *Vestfold Hjertesenter AS, Sandefjord:* J Berg-Johansen, H Tisthammer Antonsen;

Sweden

*National coordinator***:** L Jonasson; *Collaborators: A+ Science, Stockholm:* Å Ohlsson, L Bastani, T Delgado, S Gunvarsdotter, P Löf, L Persson, T Larsdotter-Damm, K Skoglund; *Capio Lund, Lund:* C Lindholm, J Thulin, E Assarsson, M Broberg; *Centralsjukhuset Kristianstad, Kristianstad*: I Torstensson, I Lager, K Hårsmar, A Knutsson; *City Heart, Stockholm:* L Hjelmaeus, R Zlatewa, E Lindemann, I McLain; *Dalecarlia Clinical RC, Rättvik:* H Larnefeldt, M Eld, M Björkman-Larnefeldt; *Hallands Sjukhus, Halmstad:* P Hårdhammar, P-A Johansson, A-C Karlsson, M Lingman, M Löfgren, A Tabandeh, L Andersson, C Palm; *Hässleholms Sjukhus, Hässleholm:* I Timberg, M Stjernberg, P Wikström; *Karolinska Universitetssjukhuset, Stockholm:* C Bergmark, O Thott, U Hedin, C Montan, O Nilsson, M Lenquist; *Linköpings Universitetssjukhus, Linköping:* L Jonasson, L Nilsson, P Wodlin, M Börjesson, A Raschberger; *Ljungby Lasarett, Ljungby:* L Ekholm, K-A Svensson, A Ågårdh, L Algotsson, M-L Bergström; *Universitetssjukhuset Mölndal, Mölndal:* F Randers, L Klintberg, U Axelsson, P-Å Moström, G Mourtzinis, P Parén, B Persson, M Risenfors, J Moodh, M Mossmark, L Ohrtgren; *Motala Lasarett, Motala:* P Ahlström, Å Törnqvist, U Rosenqvist, M Grändås, G Karlsson; *Norrlands Universitetssjukhus, Umeå:* P Ottander, A Eriksson, M Backlund, M Johansson, C Sundholm; *Öbackakliniken, Härnösand:* A Kempe, S Salomonsson, J Larsson, H Andersson, K Forsberg, A Sjödin; *Oskarshamns Sjukhus, Oskarshamn:* U Mathiesen, M Carlsson, E Keppel, K Fehling, U Robertsson; *Skellefteå Lasarett, Skellefteå:* J-H Jansson, K Boman, M Johansson, L-M Lundmark, B Norrfors; *Universitetssjukhuset, Örebro:* A Weiderman, E Jasinska, M Lundvall, K Eriksson, J Kjellberg-Eriksson, U Larsson; *Växjö Sjukhus, Växjö:* P Vasko, G Anderson, O Bergström, S Johansson, T Nyström, I Uggeldahl;

United Kingdom

National coordinator: L Bowman; *Collaborators: Aberdeen Royal Infirmary, Aberdeen:* J Webster, J Henderson, V Herd, E Wilson; *Addenbrooke's Hospital, Cambridge:* F Mir, S Blackwood (monitor), M Watts; *Barnsley District General Hospital, Barnsley:* W Khan, N Tahir, K Elliot, J Lichfield, H Marsh, M Reid; *Bedford Hospital, Bedford:* I Cooper, R de Silva, A Gallivan; *Birmingham Heartlands Hospital, Birmingham:* A Jones, L Andrews, C Jewkes; *Bradford Royal Infirmary, Bradford:* S Lindsay, K Rees, A Wilson (monitor); *Bristol Royal Infirmary, Bristol:* G Bayly, J Chambers (monitor), S George, M Halestrap; *Colwyn Bay Community Hospital, Colwyn Bay:* C Bellamy, S Evans, J James, E Pritchard, J Stockport, S Wynne; *Derriford Hospital, Plymouth:* J Fulton, J Simmonds, C Stewart, C West; *Dewsbury District Hospital, West Yorkshire:* H Chidambara, T Msimanga, B Moore, G Roberts; *Dorset County Hospital, Dorchester:* T Edwards, S Breakspear, N Fleming; *Edinburgh Royal Infirmary, Edinburgh:* D Newby, E Fraser, L Marshall, H Nailon; *Guy's Hospital, London:* J Chambers, D Parkin; *Hillingdon Hospital, Uxbridge:* M Edwards, C Mitchell, N Mahabir; *Huddersfield Royal Infirmary, Huddersfield:* H Griffiths, K Mitchell, D Appleyard, S Farr; *King's Mill Hospital, Sutton-in-Ashfield:* R Lloyd-Mostyn, S Hardingham, T Sewell; *Lister Hospital, Stevenage:* M Lynch, W Burog, M Dhaliwal, C Mfuko; *Luton and Dunstable Hospital, Luton:* C Travill, S Gent, B Norris; *Macclesfield Hospital, Macclesfield:* R Edgell, T Lake, A Taylor Bennett (monitor); *Manor Hospital, Walsall:* A Hartland, E Walton (monitor); *Memorial Hospital, Darlington:* J Murphy, G Brennan, P Cawley, L Dixon, E Rees; *Musgrove Park Hospital, Taunton:* R Andrews, T Brownlow, S Crouch, H Mills, M Nixon, N Salter; *Ninewells Hospital, Dundee:* S Pringle, S Hutcheon, H Waldie; *North Manchester General Hospital, Manchester:* J Swan, D McSorland; *North Tyneside General Hospital, North Shields:* R Curless, M Armstrong, C Ashbrook-Raby, D Bunn, R Gour, C Herriott, J James, C Robson, C Tanney, A Taylor Bennett (monitor); *Northampton General Hospital, Northampton:* P Davey, L Campey, K Smith, E Tanqueray; *Pinderfields Hospital, Wakefield:* A Munir, O Pereira, M Khalifa, B Moore; *Princess Royal Hospital, Telford:* N Capps, D Donaldson (monitor), C Miller, L Tonks; *Queen's Hospital, Burton-upon-Trent:* T Reynolds, P Basvi, J Reynolds, L Wilcox; *Queen's Medical Centre, Nottingham:* P Mansell, G Babington, E Barnes, S Beck, S Craig, L Patterson, A Selby, C Woodford; *Richard Doll Building, Oxford:* L Bowman, MJ Landray, J Armitage, H Watkins, S Beebe,

K Bowsher Brown, J James, H Lochhead, J Robertson, V Toghill, L Wright, L Young; *Rotherham District General Hospital, Rotherham:* R Muthusamy, M Lawan, C Weston; *Royal Berkshire Hospital, Reading:* W Orr, J Foxton, S Hallett, P Hilltout, L Jones, J King; *Royal Blackburn Hospital, Blackburn:* S Ramtoola, Y Grimes; *Royal Bolton Hospital, Bolton:* A Hutchesson, J Cummings, K Morris; *Royal Cornwall Hospital, Truro:* S Fleming, K Ludlow, M Parrett, S Pellow, L Quinn; *Royal Devon & Exeter Hospital, Exeter:* M James, E Green, S Keenan; *Royal United Hospital, Bath:* J Reckless, A Robinson, G Andrews, A McLenaghan; *Royal Victoria Hospital, Newcastle upon Tyne:* I ul Haq, C Albers; *Russells Hall Hospital, Dudley:* M Labib, E Higginson; *Salford Royal Hospital, Salford;* A Fitchet, E Darrel-Asherel, J Green, M Healey, K Morris, D Sexton; *Sandwell General Hospital, West Bromwich:* E Hughes, J Chackathayil, S Willetts; *Southampton General Hospital, Southampton:* C Shearman, N Pal, A Lewis, M Pasinabo, C Trevithick, D Tyler, B Watkins; *Southmead Hospital, Bristol:* M Papouchado, G Andrews, W Bertram, E Binley, S Hierons, S Kandola, C Mann, K Whitney; *St Helier Hospital, Carshalton:* H Wilcox, A Bibi, J Fuller, S Jackson; *St Mary's Hospital, Portsmouth:* P Kalra, S Howe, K Hudson, A Suttling, C Turner; *St Richard's Hospital, Chichester:* Y Wong, L Clayton-Evans, S Moore, S Stearn; *Stepping Hill Hospital, Stockport:* P Lewis, H Cochrane, J Curtis, M Holland; *Sunderland Royal Hospital, Sunderland:* S Junejo, E Dungca, T Robson, A Smith, A Taylor Bennett (monitor); *Torbay Hospital, Torquay:* C Carey, L Felmeden, A Summerhayes, J Sutton; *University Hospital of North Staffordshire, Stoke-on-Trent:* R Butler, J Creamer, J Bellaby, K Castro-Foskett, M Griffiths, J Machin, I Massey, E Sellars, J Wain*; University Hospital of Wales, Cardiff:* I McDowell, L Davies, M Davies, H Dyer, M Odam, A Waters; *University Hospital, Coventry:* M Been, V Ansell, A Campbell, D Davies, B De Burca, J Jones, A Musa; *Victoria Hospital, Blackpool:* D Roberts, R Brady, C Dickinson, L Lane, S Pickervance; *Victoria Hospital, Kirkcaldy:* M Francis, V Bryson; *Watford General Hospital, Watford:* M Clements, L Ashton, A George, K Markwell, E Walker; *West Cumberland Hospital, Whitehaven:* O Orugun, U Poultney; *Whipps Cross University Hospital, London:* F Lie, A Taneja, B Badal, V Conteh, M Jones, M Montemayor; *Worcestershire Royal Hospital, Worcester:* J Trevelyan, E Byng-Hollander, A Doughty; *Worthing Hospital, Worthing:* M Signy, A Dunne, H Fox, S Moore, S Stearn, K Wheatley; *Wycombe General Hospital, Wycombe:* S Price, N Mahabir; *Wythenshawe Hospital, Manchester:* S Ray, S Golledge, M Holland, M Murmu, A Nicholas; *Yeovil District Hospital, Yeovil:* G Brigden, J Board, C Buckley, C Vickers;

United States of America

National coordinator: CP Cannon; *Collaborators: Acadia Clinical Research LLC, Bangor, ME:* M Albert, G Baillargeon, D Harman; *Advanced Heart Care, LLC, Bridgewater, NJ:* S Mahal, J Kaur, S Padkowsky, M Walker, S Yandamuri; *Advanced Neurology Specialists, Great Falls, MT:* D Dietrich, L Armstrong, R Brown, M Casey, V Schaefer; *Albuquerque Clinical Trials, Inc., Albuquerque NM:* E Bretton, D Hsi, J Kovach, J Troy; *Asheville Cardiology Associates, Asheville, NC:* B Asbill, L Brown, T Cauthren, A Hull, O Lim, J Tompkins, J Vaughn; *Associated Cardiovascular Consultants, Voorhees, NJ:* R Perlman, D Connors, D Hoopes, D Palazzo, A Prosser, M Serrano-Rawls; *Associated Research Partners LLC, Jonesboro, AR:* B Tedder, E Johnson, T Pearson, K Rubino, P Williams; *Atlanta Heart Specialists, LLC, Cumming, GA:* N Singh, M Brown, S Dubal, E Hall, D Logwood, U Mazahir, K Raynes; *Austin Heart, PLLC, Austin, TX:* R Gammon, A Bauman, J Hatch, P Mock, N Tilton; *Austin Heart, PLLC, Jonesboro, AR:* W Abide, Jr., D Gudeman, S Minor, T Shipwash; *Aventura Heart Center, Aventura, FL:* D Korn, A Korn; *Awasty Research Network, LLC, Marion, OH:* V Awasty, E Baldwin, G Hunt, V Kaiser, C McMurray; *Bay Area Cardiology Associates P.A, Brandon, FL:* T Khan, J Al-Jumaily, T Foster, V Holbrook; *Baylor College of Medicine, Houston, TX:* V Nambi, C Ballantyne, M Jackson, P Jones, B Morris, M Techmanski, A Tran; *Beverly Hills Cardiology, Los Angeles, CA:* S Eshaghian, H Mirshkarlo; *Black Hills Cardiovascular Research, Rapid City, SD:* A Zineldine, J Bies, D Hockett, L Kimball; *Boice Willis Clinic, Rocky Mount, NC:* M Thakkar, S Varma, S Barkley-Daughtry, S Collins, S Evans-Gay, L Martin, A McKinley, L Murray, L Noel, S Prasada, R Robinson, S Wheeler; *Brigham and Women's Hospital, Boston, MA:* CP Cannon, J Andreo, S Bansilal, B Bergmark, E Bohula May, M Cavender, J Cyr, N Desai, C Fanola, N Fantony, R Giugliano, J Gutierrez, P Kazanjian, J Marti-Bernier, J Mega, R Mesa, M O'Donoghue, B Scirica, M Silverman, D Steen, L Williams; *Bryan Heart, Lincoln, NE:* C Meckel, C Orosco, R Saalfeld, N Thompson, C Wiechert; *Buffalo Medical Group, P.C., Williamsville, NY:* L Kozlowski, B Cooke, J Corbelli, A Galla,

R Stock; *Capital Cardiology Associates, Troy, NY:* R Benton, A Carroll, C Leeper, E Orvis; *Capital Cardiology Associates, Albany, NY:* P Shah, A Kasson, J Lieberman, A O'Malley, E Orvis; *Cardiology Associates of Bellin Health, Green Bay, WI:* J Rider, B Loomis, M Schantz; *Cardiology Associates of Fairfield County, P.C., Stamford, CT:* M Heiman, K Sadowski, L Scierka, J Sclafani, K Strubberg, D Mania, E Del Mastro; *Cardiology Associates of Fairfield County, P.C., Trumbull, CT:* R Jumper, K Bukoski, P Eiben, R Keegan, E Kelley, E Sekerak, J Weisberger, A Serra; *Cardiology Associates of Fairfield County, P.C., Norwalk, CT:* C Augenbraun, S Jumper, A Stuart, A Archer, T Malak, J Velky; *Cardiology Consultants, Pensacola, FL:* R Spencer, B Lane, J Lehmann; *Cardiology Research Associates, Daytona Beach, FL:* D Henderson, L Crandall, A Easterling, A Lizama, D Millard; *Cardiovascular Associates of the Delaware Valley, PA, Elmer, NJ:* M Gelernt, C Billings, D Cockrell, E Anderson; *Cardiovascular Associates of the Delaware Valley, PA, Haddon Heights, NJ:* A Pavlides, M Davis; *Cardiovascular Associates of the Delaware Valley, PA, Sewell, NJ:* D Viswanath, M Kinder, H Jeffers, S Manga, P Shaw; *Cardiovascular Associates of the Southeast, Birmingham, AL:* S Jones, T Stover; *Cardiovascular Associates of the Southeast, Birmingham, AL:* R Reeves, S Frew; *Cardiovascular Institute of the South, Opelousas, LA:* R Menuet II, K Veerina, N Domingue, L Huffman, Y Leach, T Rideaux, J Smith, L Soileau; *Cardiovascular Research Foundation of Southern California, Beverly Hills, CA:* R Karlsberg, Bhatia, A Gomez, L Levi, D Lopez; *Cardiovascular Research of Knoxville, Knoxville, TN:* C Treasure II, L Michaelis, M Parker, C Robertson, L Treasure; *CentraCare Heart and Vascular Center at St. Cloud Hospital, St. Cloud, MN:* B Erickson, A Amundson, J Humbert, H Madden; *Charles River Medical Associates, Natick, MA:* V Desai, K Lemmertz, J Zoghbi; *Charlotte Heart Group Research Center, Port Charlotte, FL:* M Malone, K Mullinax, R Schenks; *Chesapeake Cardiovascular Associates, Baltimore, MD:* D Goldscher, M Fisher, J Latteri; *Chesapeake Cardiovascular Associates, Towson, MD:* M Goldstein, H Lutz; *Chesapeake Cardiovascular Associates, Baltimore, MD:* D Peichert, E Haskel, J Powell, C Yashinski; *Clearwater Cardiovascular and Interventional Consultants, Clearwater, FL:* J Amin, D Bashton, S Burns, A Davidson, C DeSousa, C Humberger, R McGee; *Clearwater Cardiovascular and Interventional Consultants, Safety Harbor, FL:* J Zelenka, D Ferguson, C Manuel, J Quinn, J Zelik; *Cleveland Clinic Fairview Cardiovascular Medicine, Fairview Park, OH:* E Nukta, B Bittel, M Dettmer, C Palmer; *Clinical Research Associates, Florence, SC:* W Boulware, L Cooper, R Freeman; *Clinical Trials of America, Shreveport, LA:* W Zhang, K Banks, L Hall, C Hall, K Riser, S Vaz, J Winstead, L Womack; *Clinical Trials of America, Inc, Lenoir, NC:* J Dy, L Fox, E Landers, B Raby, T Whisnant; *Clinical Trials of America, INC, Hickory, NC:* S Isserman, T Annas, K Kirby, J Lail, C Moore, A Waters; *Cohen Medical Associates, Delray Beach, FL:* R Cohen, J Bossaers, L Heaney, A Hislop, L Moreiras, M Ocampo; *Community Clinical Research Center, Anderson, IN:* P Jetty, T Allen, C Custer, S Howard, A Key, S Lipps; *Comprehensive Cardiovascular Medical Group, Bakersfield, CA:* S Banerjee, S Carlos, A Garza, R Sutton; *Dayton Heart Center, Dayton, OH:* J Tobiansky, J Gluck, C Tofstad; *Doylestown Health Cardiology, a Division of Doylestown Health Physicians, Doylestown, PA:* J Kmetzo, J Brown, L Carter, R Riley, D Scott, P Seger, D Taylor, D Wood; *East Texas Cardiology, PA, Houston, TX:* A Ahmad, M Ahmed, H Ayub, S Contreras, S Iqbal, S Martinez, M Martinez; *Eastern Suffolk Cardiology, Southampton, NY:* S Donahoe, P Dalal, C Defraia, R DeStefano, S Lederman, D Lorme, M Ruhani; *Escondido Cardiology Associates, Inc, Escondido, CA:* R Acheatel, J Biggers, P Emery; *Florida Hospital, Orlando, FL:* C Kim, D Barnes, K Behm, A Dziekonski, H Karunaratne, C Stastny; *Gemini Scientific, LLC, Madison, WI:* N Bittar, S Lehmann, M Spatola, P Wilson; *Gotham Cardiovascular Research, New York, NY:* C Staniloae, E Homberg-Pinassi; *Grand View-Lehigh Valley Health Services, Buxmont Cardiology Division, Sellersville, PA:* P Hermany, K Batchlett, A Gibson, S Meissner-Dengler; *Green and Seidner Family Practice Associates, Lansdale, PA:* J Rosenfeld, B Madden, M Seidner, K Sosonkin; *Harrisonburg Medical Associates, Harrisonburg, VA:* S Pollock, S Johnson; *Health First Medical Group, Melbourne, FL:* J Salazar, R Hovland, J Jordan, S Karas, T Peacock, N Schechtmann, G Tischner, R Vicari, K Warren; *Heart and Health Institute Westside, Plantation, FL:* A Ghitis, H Cusner, M Klaus Clark; *Heart Center at St. Mark's Hospital, Salt Lake City, UT:* J Zebrack, S Christensen, C Evenson, D Fullerton; *Heart Center Research LLC, Huntsville, AL:* J Hartley, K Broadway, L Eskridge, D Raymond; *HeartCare Midwest, Peoria, IL:* T Kizhakekuttu, S Hillis, R Klundt, D McElroy; *Heritage Valley Medical Group,*

Inc, Beaver, PA.: K House, R Begg, J Acon, A Flores, J Hobbs-Williams, E Schidemantle; *HOPE Research Institute, Phoenix, AZ:* M Cooper, E Campbell, B Corcoran, S Hughes, N Miller, S Steingard; *HOPE Research Institute, Chandler, AZ:* D Einhorn, M Berry, S Dawkins Hughes, L Gilbert, E Lasala, A Loeck, N Mills, J Oppenheim; *Hudson Valley Cardiovascular Practice, P.C., Poughkeepsie, NY:* D O'Dea, S Brian, G Gerber, T Landi, J Ling, S Rimmey; *Imperial Health, LLP, Lake Charles, LA:* R Gilmore, C Bruney, E Gabbert, R Hays, L Stawecki, J Trahan, D Winey-Ward; *Indian River Medical Center – Cardiology, Vero Beach, FL:* S Baker, B Gervasio, J Labodin; *Inova Cardiology Ambulatory Research, Manassas, VA:* H Taheri, J Brooks, A Delozier, J Jayashekaramurthy, S Khachab, P Machineni, K Morgan; *Intermed, PA, Portland, ME:* C Cathcart, E Ciampanelli, W Ervin, K Soule, J Stinson; *Iowa Diabetes and Endocrinology Research Center, Des Moines, IA:* A Bhargava, L Borg, A Carver; *Jacksonville Center for Clinical Research, Jacksonville, FL:* M Koren, A West; *Kootenai Heart Clinics, LLC, Coeur d'Alene, ID:* R Jenkins, S Barnett, H Caro, J Mooney; *Kootenai Heart Clinics, LLC, Spokane, WA:* M Janout, J Bjergo, E Kelley, L Passey, K Sather; *Kore CV Research, Jackson, TN:* E Hage-Korban, M Carrington, A Childs, A Harrington, D Manns, T Phelan; *LeBauer Cardiovascular Research Foundation, Greensboro, NC:* T Stuckey, S Lord, S Milks; *Louisville Metabolic and Atherosclerosis Research Center, Louisville, KY:* H Bays, D Bushong, S Keiran, M Moore, K Weiter; *Lutherville Personal Physicians, Lutherville, MD:* F Morris, C Dignon, J Downing, D Lowry, A Metcalf; *Maine Research Associates, Auburn, ME:* E Claxton Jr., R Weiss, S Dumais; *Marin Endocrine Care & Research, Inc, Greenbrae, CA:* R Bernstein, C Singh; *McLaren Northern Michigan, Petoskey, MI:* H Colfer, A Teklinski, D Antonishen, M Antonishen, M Ronquist, C Shaw; *Medicor Cardiology, Bridgewater, NJ:* J Hall, C Hanzich; Meriter Hospital, Inc., Meriter, Madison, WI: D Lewis, A Gessler, L Skatrud; *Michigan Cardiovascular Institute, Saginaw, MI:* J Collins, V Bitzer, A Fruge, T Gauthier, M Hernandez, K Kayner, C Michon, L Naessens; *MidMichigan Medical Center Midland, Midland, MI:* W Felten, A Cryderman, M Lagalo, K Mostek, C Cluley, J Prior; *MidValley Cardiology, Kingston, NY:* E Lader, M Meyer; *Mobile Heart Specialists, PC, Mobile, AL:* C Alford, S Bryan, M Craig, J Gilley; *MODEL Clinical Research, Baltimore, MD:* P Levin, L Bromberger, D Lowry; *MultiCare Institute for Research & Innovation, Tacoma, WA:* D Guerra, P Brandon, C Burton, J Ebert, K Garrison, C Goetz, S Harris, C Lumsden, D Quinn; *MultiCare Research Institute, Tacoma, WA:* R Graf, K Garrison, S Harris, D Quinn; *National Clinical Research-Richmond, Inc, Richmond, VA:* J Scott, S Ayers, T Beasley, Finney, R Gordon, J Hoekstra, W Jeter, C Young; *Nebraska Heart Institute, Lincoln, NE:* P Dionisopoulos, C Godfrey, R Holcomb, S Krenk; *NJ Heart, Linden, NJ:* P Randhawa, S Agarwal, E Almond, E Capstraw, A Geraldo-Abache, S Kuchipudi, L Pasupuleti, C Sangiovanni, H Sheena, B Vargas; *North Alabama Research Center, LLC, Athens, AL:* E Hendrix, C Crews, J McNeese; *North Ohio Heart Center, Sandusky, OH:* M Traboulssi, A Bohn, K Humphrey, A Walton; *Northwest Heart Clinical Research, LLC, Arlington Heights, IL:* S Lupovitch, S Bellini, L Clemens, M Galindo, V Piszkiewicz, A Soni; *Norton Heart Specialists, Louisville, KY:* J Lash, T Abell, V Flanery, J Hanrahan, D Mudd; *Novant Health Heart and Vascular Institute, Charlotte, NC:* J Pasquini, V Morton, J Nikitin, P Richards, C Sander, M Voelkers; *NYU Hudson Valley Cardiology, Cortlandt Manor, NY:* G Hamroff, L Bentivenga, K Fuerst-Carter, C Hametz, L Hollenweger, C Pankovic, A Solomon; *Ocala Research Institute, INC, Ocala, FL:* R Prashad, T Colacone, M Green, P Lightcap, C McDonough, E Metivier, D Miller; *OhioHealth Research Institute, Mansfield, OH:* M Alton, D Grimwood-Fidler, G Heins, A Looney, L Orr, A Smith; *Oregon Health & Science University, Portland, OR:* W Clark, M Dolan, B Dugan, K Feest, J Foley; *Overlake Medical Clinics Cardiology, Bellevue, WA:* N Perlmutter, R Aviles, W Doucette, T Fortney, J Garceau, J Heywood, K Kanegae, C Kozlowski, D LeDoux, J Leggett, A Mahan, E McKinney, S Ostergard, J Smith, S Wagoner, S Yedinak, N Zilz; *Overlea Personal Physicians, Baltimore, MD:* B Kahn, A Campbell, V Coombs, J Phelps, E Sheridan, M Steinberg; *Palmetto Research Center, LLC, Spartanburg, SC:* R Littlefield, J Baty, A Clark, J Cooper, E Hames; *Parkview Research Center, Fort Wayne, IN:* W Collis, C Moeller, J Needham; *Pentucket Medical Associates, Haverhill, MA:* S Srivastava, S Bilazarian, C Ketis, K Roach; *Permian Research Foundation, Odessa, TX:* F Boccalandro, A Bryan; *Pottstown Medical Specialists, Inc, Pottstown, PA:* J Krantzler, N McClelland, T Muhlenberg, S Pickett; *Premier Healthcare, LLC, Bloomington, IN:* L Rink, E Anderson, A Brooks-Wolfe, B Litz, D Mobley; *Prevea Clinic, Inc., Green Bay, WI:* T Knutson,

B Belanger, P Hermans, C Quinnell; *Primary Care Cardiology Research, Inc, Ayer, MA:* T Hack, E Fisher, L Morelli, S Sullivan; *PriMed Physicians, A Member of Northeast Medical Group, Yale New Haven Health, Trumbull, CT:* C Landau, D Ferguson, T Hilts; *Providence Saint Joseph Medical Center, Burbank, CA:* D Eisenberg, G Babar, M Fam, D Fernando, D Gallegos, G Kenegos, K Reed; *Regions Hospital-Heart Center, St. Paul, MN:* M Danish Rizvi, G Erie, C Eubanks, B Foster, J Kline, W Nelson; *Research Physicians Network Alliance, Hollywood, FL:* L Tami, M Abdur Rahman, J Viera Moreno; *Research Physicians Network Alliance, Pembroke Pines, FL:* P Krichmar, J Ferreira, D Marquez, H Sanchez-Lacayo, R Yunes; *Santa Rosa Cardiology Medical Group, Inc, Santa Rosa, CA:* J Hunter, E Battistelli, T Cook, R Iverson, M Suarez; *Saratoga Cardiology Assoc., PC/Saratoga Clinical Research, LLC, Saratoga Springs, NY:* D Kandath, S Frank, G Kostedt, J Nelson; *South Florida Research Group LLC, Miami, FL:* C Hamburg, L Diaz, E Hernandez, J Roberts, K Shatsky, E Torres; *South Oklahoma Heart Research, LLC, Oklahoma City, OK:* N Tahirkheli, T Adams, K Springer, W Springer; *Southwest Florida Research, LLC, Naples, FL:* J Talano, R Ficarra, L Leo, J Nolen, M Perez, G Rappley, N Szalanski; *Southwest Heart, Tucson, AZ:* B Peart, M Ford-Tarlton, K Peart, J Stephens; *St. Johns Center for Clinical Research, Ponte Vedra, FL:* D Schlager, E Schramm, M Rabalais, C Williamson; *St. Peter's Health Partners Medical Associates - Albany Associates in Cardiology, Albany, NY:* J DeSantis, K Benedetto, E Bursey, T Harting, R Muller, R Phang, E Roccario, P Schaummann-Boyle, A Zuchelkowski; *Tallahassee Research Institute Inc, Tallahassee, FL:* J Katopodis, K Gearld, P Knap, S Liebrich; *TCR Institute, LLC, Norwalk, CT:* I Lieber, L Ferree, F Stowe, M Sutton, D Wiseman; *Tenet Florida Physician Services, Jupiter, FL:* C Vogel, R Aggarwal, C Baroni, P Beck, J Blake, E Dagher, A Gryl, M Johnson, M Smith; *The Carl & Edyth Lindner Center for Research & Education at The Christ Hospital, Cincinnati, OH:* D Kereiakes, C DeFosse, J Schwartz; *The Center for Pharmaceutical Research, P.C., Kansas City, MO:* J Ervin, S Edwards, C Gorman, A Gorsuch, S Pomeroy; *The Polyclinic, Seattle, WA:* K Huehnergarth, K Davis, M Harder, M Lim, M Schrenker, S Yedinak; *The University of Iowa, College of Public Health, Preventive Intervention Center, Iowa City, IA:* J Robinson, J Cayler, M Cherrico, D Chun-Furlong, J De La Garza; *Trinity Medical Center, Rock Island, IL:* A Pothula, C Antonio-Drabek, A Bradley, R Buresh, T Hass, C Lopez*; UCH-MHS, Colorado Springs, CO:* J Strader, Jr., A Donlin, E Ensminger, H Garcia, A Gneiting, E Graf, D Greenberg; *University of Alabama Medical Center, Birmingham, AL:* W Rogers, P Arora, T Morgan, L Saag, S Thorington; *University of Maryland, Westminster, MD:* S Jerome, L Black, A Gupta; *University of Missouri Health System, Columbia, MO:* K Aggarwal, K Belew, V Burkhardt, S Collins, S Holland Clasby, A Lau-Sieckman; *Upstate Cardiology, Greenville, SC:* J Cebe, E Calhoun, C Kissam, L Major; *Verde Valley Medical Center, Cottonwood, AZ:* S Butman, K Bescak, D Bescak, A Bigelow, T Brown, S Davidson; *Virginia Heart, Falls Church, VA:* T Haddad, T Alexander, J Jain, S McClain, T Myhera, D Overbeck, B Torre, L Wotorson; *Watson Clinic LLP, Lakeland, FL:* J Canto, C Corneal, B Donley, N McGowan, K Prisoc, M Sharrett; *Wenatchee Valley Hospital & Clinics, Wenatchee, WA:* S Kaster, J Akers, H Darlington, J Gault, J Horner, C Roozen; *Westlake Medical Research, Thousand Oaks, CA:* I Loh, R Anderson, T Call, J Esaki, P Patel, J Plocky, J Raymond, C Rideaux, L Sprafka; *Westside Center for Clinical Research, Jacksonville, FL:* M Stich, T Alexander, C Andres, C Brown, M Buda, S Ciuica, B Minker, S Perry; *York Hospital, York, PA:* K McCullum, B Doty, S Gates, K Hutcheson.

Data S2.

Supplemental Methods

Number of events (%) contributing to QoL regression analysis

	By years from latest qualifying event in study to QoL measurement at end of study			
	<1 year N(%)	1-2 years N(%)	2-3 years N(%)	>=3 years N(%)
**Myocardial infarction (n=713)**	164 (23.0)	153 (21.4)	178 (25.0)	218 (30.6)
**Coronary revascularization (urgent) (n=599)**	151 (25.2)	121 (20.3)	153 (25.5)	174 (29.0)
**Coronary revascualization (non-urgent) (n=941)**	216 (23.0)	224 (23.7)	233 (24.8)	268 (28.5)
**Non-hemorrhagic sroke (n=393)**	87 (22.1)	97 (24.7)	99 (25.2)	110 (28.0)
**Heart-failure admission (n=408)**	55 (13.5)	78 (19.1)	109 (26.7)	166 (40.7)
**Non-coronary revascularization (n=756)**	163 (21.6)	168 (22.2)	196 (25.9)	229 (30.3)
**Incident diabetes (n=778)**	209 (26.9)	179 (23.0)	194 (24.9)	196 (25.2)
**Incident cancer (n=958)**	197 (20.6)	247 (25.8)	238 (24.8)	276 (28.8)

Selection procedure for the QoL regression model

1. Covariate selection was carried out for all events with their comprehensive hierarchical temporal event history categories and the full set of participant characteristics; age and sex were retained independently of statistical significance.
2. Interactions between co-occurring qualifying events (if exceeding 5% of the number of respective contributing event) were tested and retained if significant at p<0.01.
3. Statistical difference in effects between consecutive temporal event history categories were tested in reduced model backwards (e.g. > 2 years ago, 1-2 years ago, ≤ 1 year) using F-test and p<0.01 significance level.

Selection procedure for the annual cost regression model

1. Covariate selection was carried out for all key events with their comprehensive hierarchical temporal event history categories and the full set of participant characteristics; age and sex were retained independently of statistical significance.
2. Interactions between co-occurring qualifying events (if exceeding 5% of the number of respective contributing event) were checked and retained if statistically significant at p<0.01.
3. Statistical difference in effects between consecutive temporal event history categories were tested in reduced model backwards (e.g. >2 years ago, 1-2 years ago, ≤ 1 year) using F-test and p<0.01 significance level. This procedure was carried independently for each model part in the 2-part model specifications.
4. The following tests were used to guide choice of family distribution and link function in the Generalised Linear Models (GLMs): Modified Park's test which indicates appropriateness of family distribution[41]. Pregibon's link test which checks for linearity of response on scale of estimation[42]. Hosmer-Lemeshow test which inspects whether mean prediction errors (MPEs) are significantly different from fitted values in deciles by applying F-test statistic[43]. In addition, the predictive performance of candidate models was assessed using mean prediction error (MPE), mean absolute prediction error (MAPE), and root mean square error (RMSE). Lastly, model performance was assessed using split sample cross-validation. Estimation and validation sample were repeatedly split randomly 500 times at a ratio of 2:1 considering clustering of multiple annual cost records per patient and stratifying by CVD risk[44]. Performance was separately estimated in each random sample and mean values were summarized.

Data S3.

HealthcareCost_QoL_calculator_v1.0.xlsm: An Excel program for the implementation of the HRQoL and hospital care cost models. See separate Excel file.

Table S1. QoL associated with patient characteristics, clinical factors and new adverse events (19321 contributing participants): linear regression models.

	UK perspective		US perspective	
	Mean (SE)	p-value	Mean (SE)	p-value
**Intercept**	0.327 (0.008)	<0.01	0.313 (0.008)	<0.01
**Study region**				
UK	reference		-0.023 (0.003)	<0.01
North America	0.021 (0.003)	<0.01	reference	
Other European countries	0.009 (0.003)	<0.01	-0.013 (0.003)	<0.01
Characteristics at study entry				
EQ-5D utility	0.602 (0.007)	<0.01	0.654 (0.007)	<0.01
Female	-0.025 (0.004)	<0.01	-0.022 (0.004)	<0.01
**Cardiovascular disease (ref: MI only)**				
Cerebrovascular disease (CEV) only	-0.014 (0.004)	<0.01	-0.015 (0.004)	<0.01
Peripheral artery disease (PAD) only	-0.006 (0.005)	0.29	-0.007 (0.006)	0.19
MI and CEV	-0.017 (0.005)	<0.01	-0.022 (0.005)	<0.01
MI and PAD	-0.045 (0.008)	<0.01	-0.050 (0.008)	<0.01
CEV and PAD	-0.014 (0.010)	0.18	-0.018 (0.011)	0.09
MI, CEV and PAD	-0.014 (0.014)	0.30	-0.028 (0.014)	0.05
None of MI, CEV, PAD	0.003 (0.005)	0.52	0.006 (0.005)	0.19
Atrial fibrillation	-0.016 (0.005)	<0.01	-0.016 (0.005)	<0.01
Diabetes	-0.017 (0.003)	<0.01	-0.020 (0.003)	<0.01
**Alcohol (ref: never)**				
Former	-0.007 (0.004)	0.09	-0.005 (0.004)	0.26
Current	0.008 (0.003)	<0.01	0.008 (0.003)	<0.01
**Smoking history (ref: never)**				
Former	0.001 (0.003)	0.61	0.004 (0.003)	0.12
Current	-0.023 (0.004)	<0.01	-0.022 (0.004)	<0.01
**Systolic blood pressure (mmHg) (ref: <125)**				
≥ 125 < 140	0.009 (0.003)	<0.01	0.009 (0.003)	<0.01
≥ 140	0.009 (0.003)	<0.01	0.011 (0.003)	<0.01
**Body-mass index (kg/m^2) (ref: <25)**				
≥ 25 < 30	-0.006 (0.003)	0.10	-0.005 (0.004)	0.15
≥ 30	-0.029 (0.004)	<0.01	-0.029 (0.004)	<0.01
**Glomerular filtration rate (mL/min/1.73m^2) (ref: ≥60)**				
< 45	-0.028 (0.007)	<0.01	-0.030 (0.008)	<0.01
≥ 45 <60	-0.015 (0.004)	<0.01	-0.016 (0.004)	<0.01
**Urine Albumin-to-Creatinine Ratio (UACR, mg/mmol) (ref: <3)**				
≥ 3 < 30	-0.012 (0.003)	<0.01	-0.011 (0.003)	<0.01
≥ 30	-0.032 (0.008)	<0.01	-0.031 (0.008)	<0.01
Characteristics at EQ-5D measure				
Age (centred at 67, per 10 years)	-0.020 (0.002)	<0.01	-0.024 (0.002)	<0.01
**Adverse event (vs No event)**				
**Non-haemorrhagic stroke (N=393)**				
Any history	-0.067 (0.008)	<0.01	-0.069 (0.009)	<0.01
**Heart failure admission (N=408)**				
≤1 year	-0.072 (0.014)	<0.01	-0.103 (0.015)	<0.01
1 - 2 years	-0.094 (0.015)	<0.01	-0.086 (0.016)	<0.01
>2 years	-0.013 (0.014)	0.33	-0.032 (0.013)	0.01
**Non-coronary revascularization (N=756)**				
≤1 year	-0.071 (0.011)	<0.01	-0.061 (0.013)	<0.01
1 - 2 years	-0.041 (0.012)	<0.01	-0.048 (0.012)	<0.01

>2 years	0.000 (0.009)	0.99	-0.018 (0.008)	0.03
**Incident cancer (N=958)**				
≤1 year	-0.064 (0.010)	<0.01	-0.068 (0.010)	<0.01
>1 year	-0.032 (0.006)	<0.01	-0.036 (0.006)	<0.01

Data of study participants recruited in Europe and North America was used in both analyses from the UK and US perspectives. QoL (EQ-5D utility) values for all contributing study participants were calculated using the UK or US EQ-5D value sets, depending on perspective. Event occurrence timing (≤1 year; 1-2 years and >1 or 2 years) prior to QoL measure at final follow-up visit. Models were adjusted for baseline QoL for each patient. We were unable to detect reductions in QoL associated with myocardial infarction, coronary revascularizations (urgent, non-urgent), and incident diabetes, so these events were not included in the model.
CEV, cerebrovascular disease; MI, myocardial infarction; PAD, peripheral artery disease; QoL, quality of life; SE, standard error; UK, United Kingdom; US, United States.

Table S2. Sensitivity analyses of QoL reductions associated with new adverse events estimated using only participants from UK and North America study regions, respectively.

	UK perspective (7365 UK participants)			US perspective (5193 North America participants)		
	N*	Mean (SE)	p-value	N*	Mean (SE)	p-value
**Non-haemorrhagic stroke**	**143**			**117**		
Any history		-0.101 (0.015)	<0.01		-0.055 (0.015)	<0.01
**Heart failure admission**	**88**			**185**		
≤1 year		-0.062 (0.029)	0.03		-0.039 (0.025)	0.12
1 - 2 years		-0.054 (0.032)	0.09		-0.074 (0.023)	<0.01
>2 years		-0.048 (0.035)	0.17		-0.032 (0.018)	0.08
**Non-coronary revascularization**	**217**			**289**		
≤1 year		-0.087 (0.022)	<0.01		-0.035 (0.023)	0.14
1 - 2 years		-0.037 (0.022)	0.10		-0.077 (0.019)	<0.01
>2 years		0.010 (0.018)	0.59		-0.025 (0.013)	0.06
**Incident cancer**	**383**			**283**		
≤1 year		-0.060 (0.015)	<0.01		-0.070 (0.019)	<0.01
>1 year		-0.041 (0.011)	<0.01		-0.021 (0.012)	0.07

The models with adjustments for baseline QoL for each participant (see Table S1) were re-estimated using respective UK and North America subpopulations. QoL (EQ-5D utility) values for study participants were calculated using the UK or US EQ-5D value sets, for participants in UK and North America, respectively. Event occurrence timing (≤1 year; 1-2 years and >1 or 2 years) prior to QoL measure at final follow-up visit.

*Number of study participants who experienced the respective event during the trial before QoL measure at final follow-up visit.

SE, standard error; UK, United Kingdom; US, United States.

Table S3. QoL associated with new adverse events (19321 contributing participants): scenario analyses excluding adjustments for QoL at baseline.

	UK perspective Mean (SE)	p-value	US perspective Mean (SE)	p-value
**Intercept**	0.858 (0.005)	<0.01	0.947 (0.005)	<0.01
**Study region (ref: UK/North America)**				
UK	reference		-0.064 (0.004)	<0.01
North America	0.053 (0.004)	<0.01	reference	
Other European countries	0.042 (0.003)	<0.01	-0.013 (0.004)	<0.01
Characteristics at study entry				
Female	-0.056 (0.004)	<0.01	-0.052 (0.004)	<0.01
**Cardiovascular disease (ref: MI only)**				
Cerebrovascular disease (CEV) only	-0.039 (0.005)	<0.01	-0.047 (0.005)	<0.01
Peripheral artery disease (PAD) only	-0.034 (0.006)	<0.01	-0.041 (0.007)	<0.01
MI and CEV	-0.046 (0.006)	<0.01	-0.055 (0.006)	<0.01
MI and PAD	-0.081 (0.009)	<0.01	-0.093 (0.009)	<0.01
CEV and PAD	-0.073 (0.012)	<0.01	-0.085 (0.012)	<0.01
MI, CEV and PAD	-0.065 (0.016)	<0.01	-0.084 (0.017)	<0.01
None of MI, CEV, PAD	0.000 (0.006)	0.96	0.004 (0.006)	0.48
Atrial fibrillation	-0.026 (0.006)	<0.01	-0.027 (0.006)	<0.01
Diabetes	-0.036 (0.003)	<0.01	-0.042 (0.003)	<0.01
**Alcohol (ref: never)**				
Former	-0.022 (0.005)	<0.01	-0.021 (0.005)	<0.01
Current	0.016 (0.003)	<0.01	0.018 (0.003)	<0.01
**Smoking history (ref: never)**				
Former	-0.009 (0.003)	<0.01	-0.006 (0.003)	0.05
Current	-0.051 (0.005)	<0.01	-0.052 (0.005)	<0.01
**Systolic blood pressure (mmHg) (ref: <125)**				
≥ 125 < 140	0.016 (0.003)	<0.01	0.017 (0.003)	<0.01
≥ 140	0.017 (0.003)	<0.01	0.021 (0.004)	<0.01
**Body-mass index (kg/m^2) (ref: <25)**				
≥ 25 < 30	-0.012 (0.004)	<0.01	-0.012 (0.004)	<0.01
≥ 30	-0.061 (0.004)	<0.01	-0.065 (0.004)	<0.01
**Glomerular filtration rate (mL/min/1.73m^2) (ref: ≥60)**				
< 45	-0.047 (0.009)	<0.01	-0.053 (0.009)	<0.01
≥ 45 <60	-0.025 (0.005)	<0.01	-0.029 (0.005)	<0.01
**Urine Albumin-to-Creatinine Ratio (UACR, mg/mmol) (ref: <3)**				
≥ 3 < 30	-0.017 (0.004)	<0.01	-0.019 (0.004)	<0.01
≥ 30	-0.040 (0.010)	<0.01	-0.043 (0.010)	<0.01
Characteristics at EQ-5D measure				
Age (centred at 67, per 10 years)	-0.021 (0.002)	<0.01	-0.026 (0.002)	<0.01
**Adverse event (vs No event)**				
**Non-haemorrhagic stroke (N=393)**				
Any history	-0.076 (0.010)	<0.01	-0.082 (0.010)	<0.01
**Heart failure admission (N=408)**				
≤1 year	-0.096 (0.016)	<0.01	-0.131 (0.018)	<0.01
1 - 2 years	-0.111 (0.018)	<0.01	-0.105 (0.019)	<0.01
>2 years	-0.041 (0.016)	<0.01	-0.070 (0.015)	<0.01
**Non-coronary revascularization (N=756)**				
≤1 year	-0.081 (0.013)	<0.01	-0.069 (0.016)	<0.01
1 - 2 years	-0.059 (0.014)	<0.01	-0.061 (0.015)	<0.01
>2 years	-0.031 (0.011)	<0.01	-0.054 (0.010)	<0.01
**Incident cancer (N=958)**				

≤1 year	-0.057 (0.011)	<0.01	-0.062 (0.012)	<0.01
>1 year	-0.034 (0.007)	<0.01	-0.036 (0.008)	<0.01

QoL (EQ-5D utility) associated with adverse events (19321 contributing participants) in UK and US perspective: scenario analyses excluding adjustments for QoL at baseline prior to estimation. CEV, cerebrovascular disease; MI, myocardial infarction; PAD, peripheral artery disease; QoL, quality of life; SE, standard error; UK, United Kingdom; US, United States.

Table S4. QoL associated with patient characteristics, clinical factors and new adverse events in China: a scenario analysis using the 7778 participants recruited in China.

	Adjusted for baseline QoL		Not adjusted for baseline QoL	
	Mean (SE)	p-value	Mean (SE)	p-value
**Intercept**	0.351 (0.017)	<0.01	0.962 (0.003)	<0.01
Characteristics at study entry				
EQ-5D utility	0.625 (0.017)	<0.01	-	
Female	-0.020 (0.004)	<0.01	-0.034 (0.004)	<0.01
**Cardiovascular disease (ref: MI only)**				
Cerebrovascular disease (CEV) only	-0.048 (0.005)	<0.01	-0.088 (0.005)	<0.01
Peripheral artery disease (PAD) only	0.028 (0.031)	0.36	0.020 (0.033)	0.55
MI and CEV	-0.020 (0.005)	<0.01	-0.038 (0.006)	<0.01
MI and PAD	-0.029 (0.028)	0.30	-0.040 (0.030)	0.19
CEV and PAD	0.048 (0.038)	0.20	0.002 (0.041)	0.96
MI, CEV and PAD	-0.016 (0.043)	0.71	-0.084 (0.047)	0.07
None of MI, CEV, PAD	0.000 (0.005)	0.99	-0.003 (0.005)	0.53
**Body-mass index (kg/m^2) (ref: <25)**				
≥ 25 < 30	0.003 (0.003)	0.40	0.001 (0.004)	0.85
≥ 30	-0.020 (0.005)	<0.01	-0.028 (0.006)	<0.01
**Glomerular filtration rate (mL/min/1.73m^2) (ref: ≥60)**				
< 45	-0.049 (0.015)	<0.01	-0.062 (0.016)	<0.01
≥ 45 <60	-0.035 (0.009)	<0.01	-0.048 (0.009)	<0.01
**Urine Albumin-to-Creatinine Ratio (UACR, mg/mmol) (ref: <3)**				
≥ 3 < 30	-0.005 (0.005)	0.25	-0.016 (0.005)	<0.01
≥ 30	-0.035 (0.010)	<0.01	-0.049 (0.011)	<0.01
Characteristics at EQ-5D measure				
Age (centred at 67, per 10 years)	-0.029 (0.002)	<0.01	-0.036 (0.002)	<0.01
**Adverse event (vs No event)**				
**Myocardial infarction (N=304)**				
Any history	-0.037 (0.010)	<0.01	-0.039 (0.010)	<0.01
**Coronary revascularization (urgent) (N=234)**				
Any history	0.029 (0.011)	<0.01	0.027 (0.012)	0.02
**Non-haemorrhagic stroke (N=276)**				
≤1 year	-0.163 (0.016)	<0.01	-0.164 (0.017)	<0.01
1 - 2 years	-0.131 (0.018)	<0.01	-0.136 (0.019)	<0.01
>2 years	-0.071 (0.012)	<0.01	-0.074 (0.013)	<0.01
**Heart failure admission (N=321)**				
≤1 year	-0.111 (0.013)	<0.01	-0.130 (0.014)	<0.01
>1 year	-0.024 (0.010)	0.02	-0.041 (0.011)	<0.01
**Incident cancer (N=139)**				
≤1 year	-0.152 (0.019)	<0.01	-0.159 (0.021)	<0.01
>1 year	-0.012 (0.015)	0.41	-0.015 (0.016)	0.34

7778 from the 8629 participants recruited in China provided QoL (EQ-5D) measure at end of follow-up and were included in this analysis.

The model with adjustment for QoL at baseline was estimated using the same strategy used for main analyses. The model without adjustment for QoL at baseline used the same covariates as the final model with adjustment for QoL at baseline and then excluded the baseline QoL adjustment prior to estimation.

CEV, cerebrovascular disease; MI, myocardial infarction; PAD, peripheral artery disease; QoL, quality of life; SE, standard error.

Table S5. Goodness of fit tests of Generalised Linear Model specifications for models of annual hospital costs from the UK and US healthcare perspectives.

Generalised Linear Model Distribution/ link function	p-value for Hosmer-Lemeshow test (observed vs predicted mean error deviation in deciles)	p-value for Pregibon's test (Link test for response linearity on estimation scale)	Mean error	Mean absolute error	Root mean squared error
**UK healthcare perspective**					
**Single equation**					
Gaussian/ Identity	<0.01	0.04	-1.0	753.9	2112.6
Gaussian/ Ln	<0.01	<0.01	122.2	926.9	2382.6
Poisson/ Ln	<0.01	<0.01	14.7	821.7	4424.4
Gamma/Ln	0.34	<0.01	1150.5	1860.4	144229.6
**Two-part equation**					
Gaussian/ Identity	0.23	<0.01	-1.1	742.4	2115.9
Gaussian/ Ln	<0.01	<0.01	2.2	754.1	2128.6
Poisson/ Identity	0.94	0.03	-1.0	743.7	2116.3
Poisson/ Ln	0.01	<0.01	0.1	747.1	2129.0
Gamma/ Identity	**0.78**	**0.61**	**-1.4**	**744.1**	**2117.9**
Gamma/ Ln	<0.01	<0.01	7.2	748.0	2146.3
**US Medicare perspective**					
**Single equation**					
Gaussian/ Identity	<0.01	0.81	-2.5	2417.1	5002.2
Gaussian/ Ln	<0.01	<0.01	431.0	3011.6	5698.4
Poisson/ Ln	<0.01	<0.01	27.6	2685.9	9142.0
Gamma/Ln	<0.01	<0.01	1811.9	4163.4	152990.1
**Two-part equation**					
Gaussian/ Identity	<0.01	<0.01	-2.7	2387.2	5015.5
Gaussian/ Ln	<0.01	<0.01	10.0	2410.0	5026.2
Poisson/ Identity	0.02	<0.01	-2.7	2391.9	5017.0
Poisson/ Ln	0.06	<0.01	-1.2	2396.8	5031.7
Gamma/ Identity	**0.04**	**0.03**	**-6.9**	**2392.4**	**5021.0**
Gamma/ Ln	0.04	<0.01	4.2	2396.0	5041.0

The Modified Park Test slope coefficients indicate the appropriate family distribution (0 = Gaussian, 1 = Poisson, 2=Gamma, 3 = Inverse Gaussian). The coefficients were 1.5 and 1.8 for UK and US cost regression models, respectively, indicating Gamma family distribution was appropriate. The selected models are shaded. LN, natural logarithm; UK, United Kingdom; US, United States.

Table S6. Annual hospital costs from UK healthcare perspective: two-part regression model.

	Part 1: Likelihood of incurring hospital costs in year Logistic model		Part 2: Annual hospital cost (£), if any incurred GLM with Gamma distribution and identity link	
	Estimate (SE)	p-value	Estimate (SE)	p-value
**Intercept**	-1.87 (0.04)	<0.01	1601 (49)	<0.01
**BASELINE CHARACTERISTICS**				
Female (ref: male)	0.20 (0.03)	<0.01	-12 (71)	0.87
**Region (ref: UK)**				
North America	-0.49 (0.03)	<0.01	172 (64)	<0.01
Other European countries	-0.30 (0.03)	<0.01	618 (69)	<0.01
**Diastolic blood pressure (mmHg) (ref: ≥ 75 < 85)**				
<75	0.10 (0.03)	<0.01		
≥ 85	-0.03 (0.03)	0.24		
**Previous disease**				
**Cardiovascular disease (ref: MI only)**				
Cerebrovascular disease (CEV) only	0.11 (0.04)	<0.01	162 (87)	0.06
Peripheral artery disease (PAD) only	0.10 (0.05)	0.04	326 (119)	<0.01
MI and CEV	0.24 (0.04)	<0.01	-91 (89)	0.31
MI and PAD	0.29 (0.06)	<0.01	266 (177)	0.13
CEV and PAD	0.18 (0.08)	0.03	577 (249)	0.02
MI, CEV and PAD	0.35 (0.11)	<0.01	45 (195)	0.82
No MI, CEV nor PAD	0.08 (0.04)	0.08	-132 (102)	0.19
Heart failure	0.21 (0.05)	<0.01	471 (143)	<0.01
Atrial fibrillation	0.34 (0.04)	<0.01	401 (120)	<0.01
Prior diabetes	0.16 (0.03)	<0.01	198 (71)	<0.01
**Medication use (vs none)**				
Amiodarone	0.30 (0.08)	<0.01		
**Smoking status (ref: never)**				
Former	0.09 (0.02)	<0.01		
Current	0.07 (0.04)	0.08		
**Alcohol status (ref: never)**				
Former	0.10 (0.04)	0.01		
Current	-0.08 (0.03)	<0.01		
**Body-mass index (kg/m2) (ref: <25)**				
≥ 25 < 30	0.03 (0.03)	0.30		
≥ 30	0.17 (0.03)	<0.01		
**Glomerular filtrate rate (mL/min/1.73m2) (ref: ≥60)**				
<45	0.21 (0.06)	<0.01	295 (147)	0.05
≥45 <60	0.08 (0.04)	0.04	275 (102)	<0.01
**Urine Albumin-to-Creatinine Ratio (UACR, mg/mmol) (ref: <3)**				
≥ 3 < 30	0.14 (0.03)	<0.01		
≥ 30	0.28 (0.07)	<0.01		
**TIME-UPDATED COVARIATES**				
Current Age (centred at 67 years, per 10 years)	0.17 (0.02)	<0.01	116 (33)	<0.01
**Within-trial adverse events (vs none)**				
**Myocardial infarction (MI) (N=989)**				
≤1 year	7.43 (1.00)	<0.01	943 (205)	<0.01

>1 year	0.56 (0.10)	<0.01	694 (270)	0.01
**Coronary revascularization (urgent) (N=735)**				
≤1 year*			2983 (226)	<0.01
>1 year	0.20 (0.11)	0.08	177 (256)	0.49
**Coronary revascularization (non-urgent) (N=1047)**				
≤1 year*			2954 (161)	<0.01
>1 year	0.43 (0.07)	<0.01	646 (204)	<0.01
**Non-haemorrhagic stroke (N=549)**				
≤1 year	4.68 (0.23)	<0.01	1612 (292)	<0.01
>1 year	0.56 (0.09)	<0.01	432 (277)	0.12
**Heart failure admission (N=647)**				
≤1 year*				
>1 year	0.99 (0.08)	<0.01		
Any history			1536 (237)	<0.01
**Non-coronary revascularization (N=901)**				
≤1 year*			4146 (201)	<0.01
>1 year	0.61 (0.07)	<0.01	1330 (226)	<0.01
**Incident diabetes (N=840)**				
Any history	0.27 (0.06)	<0.01		
**Incident cancer (N=1618)**				
≤1 year	3.52 (0.08)	<0.01		
1 - 2 years	0.83 (0.07)	<0.01		
>2 years	0.53 (0.08)	<0.01		
Any history	-		1189 (117)	<0.01
**Vascular death (≤1 year) (N=708)**	1.32 (0.10)	<0.01	844 (353)	0.02
**Nonvascular death (≤1 year) (N=960)**	1.70 (0.09)	<0.01	1583 (218)	<0.01
**Proportion of year censored (%)†**	-1.80 (0.05)	<0.01	-688 (129)	<0.01
Events' interactions (≤1 year) ‡				
**MI * vascular death (N=109)**	-6.59 (1.05)	<0.01	-1570 (546)	<0.01
**Non-haemorrhagic stroke * vascular death (N=43)**	-2.37 (0.66)	<0.01		
**Incident cancer * Nonvascular death (N=288)**	-1.51 (0.24)	<0.01		

Data of study participants recruited in Europe and North America was used in the analysis from the UK perspective. Only hospital inpatient care was included and all hospital admissions were costed using UK National Health Service or Medicare program reference costs, respectively.
Hospital admissions were costed using UK National Health Service reference cost.
*Heart failure admission, coronary (urgent and non-urgent) and non-coronary revascularization events had 100% probability of hospital costs in year of event. Years with these events were, therefore, excluded from part 1 modelling of probability and probability of 100% should be given to these 3 events for incurring hospital costs in year of event.
† Proportion (%) of final year in study not fully observed due to end of study.
‡ Statistically significant interaction (p<0.01) was observed between the two events of interest in the cost regression model.
CEV, cerebrovascular disease; GLM, generalised linear model; MI, myocardial infarction; PAD, peripheral artery disease; SE, standard error; UK, United Kingdom.

Table S7. Annual hospital costs from US healthcare perspective: two-part regression model.

	Part 1: Likelihood of incurring hospital costs in year Binomial logistic model		Part 2: Annual hospital cost ($), if any incurred GLM with Gamma distribution and identity link	
	Estimate (SE)	p-value	Estimate (SE)	p-value
**Intercept**	-2.36 (0.04)	<0.01	7899 (146)	<0.01
**BASELINE CHARACTERISTICS**				
Female (ref: male)	0.20 (0.03)	<0.01	-220 (147)	0.14
**Region (ref: North America)**				
UK	0.49 (0.03)	<0.01	58 (145)	0.69
Other European countries	0.19 (0.03)	<0.01	-380 (160)	0.02
**Diastolic blood pressure (mmHg) (ref: ≥ 75 < 85)**				
<75	0.10 (0.03)	<0.01	408 (129)	<0.01
≥ 85	-0.03 (0.03)	0.24	-86 (155)	0.58
**Previous disease**				
**Cardiovascular disease (ref: Myocardial infarction (MI) only)**				
Cerebrovascular disease (CEV) only	0.11 (0.04)	<0.01		
Peripheral artery disease (PAD) only	0.10 (0.05)	0.04		
MI and CEV	0.24 (0.04)	<0.01		
MI and PAD	0.29 (0.07)	<0.01		
CEV and PAD	0.18 (0.08)	0.04		
MI, CEV and PAD	0.35 (0.11)	<0.01		
No MI, CEV nor PAD	0.08 (0.04)	0.08		
Heart failure	0.21 (0.05)	<0.01	2081 (370)	<0.01
Atrial fibrillation	0.34 (0.04)	<0.01	756 (261)	<0.01
Prior diabetes	0.16 (0.03)	<0.01		
**Medication use (vs none)**				
Amiodarone	0.30 (0.08)	<0.01	1932 (593)	<0.01
**Smoking status (ref: never)**				
Former	0.10 (0.02)	<0.01		
Current	0.07 (0.04)	0.07		
**Alcohol status (ref: never)**				
Former	0.10 (0.04)	<0.01		
Current	-0.08 (0.03)	<0.01		
**Body-mass index (kg/m^2) (ref: <25)**				
≥ 25 < 30	0.03 (0.03)	0.30		
≥ 30	0.17 (0.03)	<0.01		
**Glomerular filtrate rate (mL/min/1.73m^2) (ref: ≥60)**				
<45	0.21 (0.06)	<0.01		
≥45 <60	0.08 (0.04)	0.04		
**Urine Albumin-to-Creatinine Ratio (UACR, mg/mmol) (ref: <3)**				
≥ 3 < 30	0.14 (0.03)	<0.01		
≥ 30	0.28 (0.07)	<0.01		
**TIME-UPDATED COVARIATES**				
**Current Age (centred at 67 years , per 10 years)**	0.17 (0.02)	<0.01	-14 (65)	0.83
**Within-trial adverse events (vs none)**				

**MI (N=989)**				
≤1 year	7.43 (1.01)	<0.01	1424 (396)	<0.01
>1 year	0.56 (0.10)	<0.01	619 (499)	0.22
**Coronary revascularization (CRV) (urgent) (N=735)**				
≤1 year[*]			8748 (487)	<0.01
>1 year	0.20 (0.11)	0.08	1754 (749)	0.02
**CRV (non-urgent) (N=1047)**				
≤1 year[*]			10995 (323)	<0.01
>1 year	0.43 (0.07)	<0.01	2540 (551)	<0.01
**Non-haemorrhagic stroke (N=549)**				
≤1 year	4.68 (0.23)	<0.01		
>1 year	0.56 (0.09)	<0.01		
**Heart failure admission (N=647)**				
≤1 year[*]				
>1 year	0.99 (0.09)	<0.01		
Any history			3429 (485)	<0.01
**Non-coronary revascularization (N=901)**				
≤1 year[*]			7630 (350)	<0.01
>1 year	0.61 (0.07)	<0.01	3249 (382)	<0.01
**Incident diabetes (N=840)**				
Any history	0.27 (0.06)	<0.01		
**Incident cancer (N=1618)**				
≤1 year	3.52 (0.08)	<0.01		
1 - 2 years	0.83 (0.08)	<0.01		
>2 years	0.53 (0.08)	<0.01		
Any history			3926 (326)	<0.01
**Vascular death (≤1 year) (N=708)**	1.32 (0.10)	<0.01		
**Nonvascular death (≤1 year) (N=960)**	1.70 (0.09)	<0.01		
**Proportion of year censored (%)**[†]	-1.80 (0.05)	<0.01	-2321 (276)	<0.01
***Event interactions* (≤1 year)** [‡]				
**MI p<0.05 CRV (urgent) (N=507)**			7953 (787)	<0.01
**MI p<0.05 CRV (non-urgent) (N=71)**			8779 (1993)	<0.01
**MI p<0.05 vascular death (N=109)**	-6.59 (1.05)	<0.01		
**Non-haemorrhagic stroke p<0.05 vascular death (N=43)**	-2.36 (0.66)	<0.01		
**Incident cancer * Nonvascular death (N=288)**	-1.51 (0.24)	<0.01		

Data of study participants recruited in Europe and North America was used in the analysis from the US perspective. Only hospital inpatient care was included and all hospital admissions were costed using the Medicare program reference cost.

[*] Heart failure admission, coronary (urgent and non-urgent) and non-coronary revascularization events had 100% probability of hospital costs in year of event. Years with these events were, therefore, excluded from part 1 modelling of probability and probability of 100% should be given to these 3 events for incurring hospital costs in year of event.

[†] Proportion (%) of final year in study not fully observed due to end of study.

[‡] Statistically significant interaction (p<0.01) was observed between the two events of interest in the cost regression model.

CEV, cerebrovascular disease; CRV, coronary revascularisation procedure; GLM, generalised linear model; MI, myocardial infarction; PAD, peripheral artery disease; SE, standard error; UK, United Kingdom.

Table S8. Sensitivity analysis on additional annual hospital costs associated with new adverse events using only UK and North America participant data.

Adverse event	N*	Additional hospital costs (95% CI)		N*		
		UK region, UK£ (8,381 participants; 34,996 annual periods)			North America region, US$ (6082 participants; 29,011 annual periods)	
Non-fatal MI with CRV (urgent) †	125			166		
<1 year		6224	(5136, 7772)		25682	(24271, 27127)
≥1 year		656	(236, 1221)		997	(377, 1751)
Non-fatal MI without CRV (urgent) †	164			121		
<1 year		2600	(1935, 3449)		9037	(7579, 10702)
≥1 year		628	(274, 1010)		1000	(379, 1754)
Fatal MI	48			31		
<1 year		393	(-338, 1254)		5218	(2251, 9802)
CRV (urgent) without MI[2]	43			114		
<1 year		5019	(3935, 6455)		15898	(14525, 17431)
≥1 year		8	(-216, 347)		357	(-142, 925)
CRV (non-urgent) without MI[2]	197			423		
<1 year		5662	(4955, 6416)		17312	(16438, 18330)
≥1 year		188	(1, 430)		1150	(624, 1720)
Non-fatal non-haemorrhagic stroke	197			152		
<1 year		3710	(2875, 4658)		6022	(5550, 6511)
≥1 year		459	(161, 823)		356	(-37, 766)
Fatal non-haemorrhagic stroke	18			15		
<1 year		2507	(1181, 4471)		4257	(800, 6969)
Heart failure admission	138			306		
<1 year		3095	(2455, 3752)		10924	(9601, 12432)
≥1 year		959	(618, 1359)		2354	(1681, 3134)
Non-coronary revascularization	255			350		
<1 year		5490	(4750, 6338)		13729	(12838, 14737)
≥1 year		660	(368, 994)		1183	(657, 1727)
Non-fatal incident cancer	544			395		
≤1 year		2528	(2185, 2878)		6505	(5753, 7274)
1 - 2 years		640	(460, 843)		2045	(1496, 2645)
>2 years		656	(460, 875)		1177	(649, 1758)
Incident diabetes	286			275		
Any history		27	(-29, 90)		311	(45, 568)
Fatal incident cancer	112			94		
<1 year		1475	(923, 2129)		4026	(2475, 5813)
Vascular death without MI and without non-haemorrhagic stroke	180			187		
<1 year		643	(167, 1348)		1910	(1342, 2539)
Nonvascular death without incident cancer	163			178		

<1 year	1569	(1175, 2007)	2065	(1535, 2600)

Only hospital inpatient care included. Hospital admissions of participants from Europe and North America, respectively, were costed using UK National Health Service or Medicare program reference costs, respectively.

[*]Number of people who experienced the respective event after entry into trial

[†]Significant interaction between MI and CRV (urgent) ($p<0.01$) observed only for US costs. Other interactions (MI and vascular death, non-haemorrhagic stroke and vascular death and incident cancer and non-vascular death) observed for both UK and US annual hospital costs.

CI, confidence interval; CRV, coronary revascularisation procedure; MI, myocardial infarction; UK, United Kingdom.

Table S9. Comparison of quality of life reductions associated with new cardiovascular events in people with history of cardiovascular disease.

Study	REVEAL	ODYSSEY OUTCOMES	HPS2-THRIVE	VALIANT
Author, publication year	Lui et al., 2023 (this study)	Bhatt et al., 2020[34]	Kent et al., 2016[23]	Lewis et al., 2014[3]
Study type	RCT	RCT	RCT	RCT
Population	History of atherosclerotic CVD	1-12 months post acute coronary syndrome	History of cardiovascular disease	Within 10 days post acute MI and with heart failure
No. of participants	30449	18924	25673	14703
Number of QoL measurements in study	2	>2	1	>2
Years of QoL surveys	2011-2017	2012-2018	2007-2010	1998-2003
QoL instrument, valuation	EQ-5D-5L, UK/US	EQ-5D-3L, US	EQ-5D-3L, UK	EQ-5D-3L, UK/US
Statistical model	OLS, adjusted (including baseline QoL)	Mixed effects, adjusted (including baseline QoL)	OLS, adjusted	Mixed effects, adjusted (including QoL before event)
**Cardiovascular event** (any)	NA	NA	NA	UK: 0.08 (0.04, 0.11)<br>US: 0.07 (0.05, 0.10)
**MI** (any)	Reported "No change"			UK: 0.06 (0.01, 0.11)<br>US: 0.06 (0.02, 0.10)
<=1yr		≤90 days post event: 0.028 (0.021, 0.034)	0.049 (NS)	
>1yr		>90 days post event: 0.023 (0.018, 0.027)	0.002 (NS)	
**Stroke** (any)	Non-haemorrhagic only:<br>UK: 0.067 (0.05, 0.083)<br>US: 0.069 (0.53, 0.086)	Ischemic only:		UK: 0.18 (0.08, 0.28)<br>US: 0.15 (0.07, 0.22)
<=1yr		≤90 days post event: 0.073 (0.056, 0.090)	0.136 (0.055, 0.217)	
>1yr		>90 days post event: 0.069 (0.059, 0.080)	0.118 (0.077, 0.160)	

**Coronary revascularization** (any)	Reported “No change”			NA
<=1yr		≤90 days post event: 0.007 (0.001, 0.014)	-0.003 (NS)	
>1yr		>90 days post event: -0.002 (-0.007, 0.002)	-0.023 (NS)	
**Heart failure admission** (any)				UK: 0.05 (0.01, 0.10)<br>US: 0.05 (0.01, 0.08)
<=1yr	UK: 0.072 (0.046, 0.099)<br>US: 0.103 (0.074, 0.132)		0.129 (0.054, 0.203)	
1-2 yrs/ >1yr	UK: 0.094 (0.064, 0.123)<br>US: 0.086 (0.056, 0.117)		0.083 (0.029, 0.136)	
> 2 yrs	UK: 0.013 (NS)<br>US: 0.032 (0.007, 0.056)			
**Non-coronary revascularization** (any)				
<=1yr	UK: 0.071 (0.050, 0.093)<br>US: 0.061 (0.035, 0.087)		0.083 (0.036, 0.129)	
1-2 yrs	UK: 0.041 (0.017, 0.064)<br>US: 0.048 (0.023, 0.072)		0.022 (NS)	
> 2 yrs	UK: 0.000 (NS)<br>US: -0.018 (0.001, 0.035)			
**Angina admission (any)**	NA		NA	NA

<=1yr		≤90 days post event: 0.021 (0.003, 0.045)		
>1yr		>90 days post event: -0.001 (-0.014, 0.016)		
**Resuscitated sudden death (any)**	NA	NA	NA	UK: 0.22 (NS)<br>US: 0.25 (NS)
**Incident diabetes** (any)	Reported “No change”	NA	NA	NA
**Incident cancer** (any)		NA	NA	NA
<=1yr	UK:0.064 (0.044, 0.083)<br>US: 0.068 (0.048, 0.088)			
>1yr	UK: 0.032 (0.019, 0.044)<br>US: 0.036 (0.023, 0.048)			
**Other nonvascular events reported**			Diabetes complication, musculoskeletal, gastrointestinal bleed, other GI event, bleed (non GI or stroke), infection, skin event	

CVD, cardiovascular disease; HPS2-THRIVE, The Heart Protection Study 2–Treatment of HDL to Reduce the Incidence of Vascular Events; MI, myocardial infarction; NS, not statistically significant; NA, not available; OLS, ordinary least square; QoL, quality of life; RCT, randomised controlled trial; REVEAL, Randomized Evaluation of the Effects of Anacetrapib through Lipid Modification trial; UK, United Kingdom; US, United States; VALIANT, Valsartan in Acute Myocardial Infarction trial.
